# Significance of Serum-Plasma Leptin Profile during Pregnancy in Gestational Diabetes Mellitus: A Systematic Review and Meta-Analysis

**DOI:** 10.3390/jcm11092433

**Published:** 2022-04-26

**Authors:** María del Mar Roca-Rodríguez, Pablo Ramos-García, Cristina López-Tinoco, Manuel Aguilar-Diosdado

**Affiliations:** 1 Department of Endocrinology and Nutrition and Biomedical Research and Innovation Institute of Cadiz (INiBICA), Puerta del Mar University Hospital, 11009 Cadiz, Spain; cristinalopeztinoco@gmail.com (C.L.-T.); manuel.aguilar.sspa@juntadeandalucia.es (M.A.-D.); 2 Department of Oral Medicine, School of Dentistry, University of Granada, 18071 Granada, Spain; 3 Department of Medicine, Cadiz University (UCA), 11003 Cadiz, Spain

**Keywords:** gestational diabetes mellitus, leptin, plasma/serum, materno-fetal outcomes, systematic review, meta-analysis

## Abstract

Gestational diabetes mellitus (GDM) represents a stage of subclinical inflammation and a risk factor for subsequent future type 2 diabetes and cardiovascular disease development. Leptin has been related with vascular and metabolic changes in GDM with heterogeneous and contradictory results with respect to their possible involvement in maternal, perinatal, and future complications. Our objective is to evaluate current evidence on the role of leptin in maternal and perinatal complications in women with GDM. PubMed, Embase, Web of Science, and Scopus databases were searched. We evaluated the studies’ quality using the Newcastle-Ottawa scale. Meta-analyses were conducted, and heterogeneity and publication bias were examined. Thirty-nine relevant studies were finally included, recruiting 2255 GDM and 3846 control pregnant women. Leptin levels were significantly higher in GDM participants than in controls (SMD = 0.57, 95%CI = 0.19 to 0.94; *p* < 0.001). Subgroup meta-analysis did not evidence significant differences in leptin in the different trimesters of pregnancy. Meta-regression showed a positive significant relationship for HOMA in the GDM group (*p* = 0.05). According to these results, it seems that high levels of leptin can be used as predictive markers in GDM.

## 1. Introduction

Gestational diabetes mellitus (GDM) is defined as carbohydrate intolerance that begins, or is first recognized, during pregnancy. It complicates about 1–14% of all pregnancies worldwide [1]. GDM mothers are at increased risk of prenatal morbidity and type 2 diabetes mellitus (T2DM) after pregnancy, and their offspring are more likely to be obese and have impaired glucose tolerance and T2DM in their early adulthood [2]. In GDM, complications can affect both mother and fetus. In infants, GDM is the main cause of macrosomia, as a result of maternal hyperglycemia and increased transport of glucose, amino acids, and fatty acids through the placenta that increase endogenous fetal IGF-1 production. That premature increase in fetal insulin production may cause stress on beta-pancreatic cells, leading to their dysfunction and insulin resistance. The risk of shoulder dystocia, prolonged labor, postpartum hypoglycemia leading to brain damage, and metabolic diseases are also increased [3]. Mothers with GDM are at increased risk of complications such as preterm birth, preeclampsia, and instrumental delivery [4].

It has been established that adipokines play a key role in the regulation of many crucial processes in the human body, such as glucose and lipid metabolism, insulin sensitivity, appetite, immune response, and inflammation, and may be treated as potential targets for novel therapeutic strategies in numerous medical conditions [5]. Leptin is a satiety-controlling hormone released mainly by adipocytes in response to adequate energy stores, decreasing appetite by hypothalamic stimulation of anorexigenic peptides. During pregnancy, the placenta becomes the main producer of leptin, increasing its endogenous production, favoring placental passage of amino acids to the fetus [3]. Overall, the baseline concentration of circulating leptin is higher in GDM patients, but a smaller increase in the levels of this protein can be observed throughout the course of pregnancy [5]. Elevated leptin levels may contribute to fetal macrosomia, given the hyperleptinemia present in obese and GDM states [3]. In addition, leptin has been associated with the process of placental neoformation functioning as a growth, angiogenic, and immunomodulatory factor [6]. Although there have been a number of human studies on leptin and GDM during recent decades, inferences have been hindered due to significant heterogeneities in these studies concerning design, population characteristics, assay methods, timing of blood sample collection, and definition/diagnosis of GDM [7], and results on circulating leptin in patients with GDM have been inconsistent. Maternal leptin levels appeared increased in women with GDM in most studies, while in other studies no significant variations were detected [8].

The purpose of this systematic review and meta-analysis is to define the current knowledge on maternal circulating leptin levels during pregnancy in GDM, as a biochemical mediator associated with its pathophysiology and their potential use as risk markers for GDM development, and to identify important evidence gaps.

## 2. Material and Methods

The present systematic review and meta-analysis was reported complying with *Meta-analysis of Observational Studies in Epidemiology* (MOOSE) guidelines and *Preferred Reporting Items for Systematic Reviews and Meta-Analyses* (PRISMA) [9,10], and was designed and conducted closely following the criteria of *Cochrane Collaboration* [11].

### 2.1. Protocol

A study methodological protocol was a priori registered in PROSPERO international prospective register of systematic reviews, publicly available (www.crd.york.ac.uk/PROSPERO, accessed and registered on 26 July 2020; registration code CRD42020194274 [12]), for the purpose of minimizing the risk of bias of our study, offering better transparency, precision, and integrity. The protocol also followed the PRISMA-P statement in order to ensure a rigorous reporting approach [13].

### 2.2. Search Strategy

We searched MEDLINE/PubMed, Embase, Scopus, and Web of Science databases for studies published before April-2021, with no lower date limit. Searches were conducted by combining thesaurus terms (i.e., MeSH and EMTREE) with free terms (Table S1), designed and built in order to maximize sensitivity. We also hand searched the reference lists of retrieved studies for additional target studies. All references were managed using Mendeley Desktop v.1.19.8 (Elsevier, Amsterdam, The Netherlands); duplicate references were eliminated using this reference manager.

### 2.3. Eligibility Criteria

Inclusion criteria: (1) original research from primary-level studies without publication language or date, follow up periods, geographical area or age restrictions; (2) GDM subjects compared to pregnant women without GDM as control group; (3) leptin levels evaluation from maternal plasma or serum; (4) observational study design, regardless of its cross-sectional/longitudinal study design or prospective/retrospective nature; (5) the name of authors, affiliations, clinical settings and recruitment periods were gathered and analyzed in order to detect potential overlapping populations. In such cases, the most recent studies or those reporting more complete datasets were included.

Exclusion criteria: (1) retracted articles, interventional studies, reviews, meta-analyses, case reports, editorials, letters, abstracts of scientific meetings, personal opinions or comments and book chapters; (2) in vitro and animal experimental studies; (3) studies that do not assess the disease of interest (i.e., GDM), do not study leptin levels, or those without a control group; (4) studies reporting insufficient data to extract or estimate mean ± standard deviation (SD); (5) data from overlapping populations.

### 2.4. Study Selection Process

Primary-level studies were selected in two phases, first screening the titles and abstracts of retrieved papers, and then reading the full text selected articles, excluding those that did not meet our precedent review eligibility criteria. Eligibility criteria were applied by two authors (MMRR and CLT) in an independent manner. Discrepancies were resolved by consensus with a third author (PRG). 

### 2.5. Data Extraction

Two authors (MMRR and CLT) independently extracted data from the selected articles, completing a data collection form in a standardized manner using Excel v. Microsoft Office Professional Plus 2013 (Microsoft, Redmond, WA, USA). These data were additionally cross-checked in multiples rounds, solving discrepancies by consensus. Data were gathered on the first author, publication year, study country and continent, language, sample size, source of sample (i.e., plasma or serum), leptin determination—extracting means ± SD, measuring units, technique and proper quantification—in GDM and controls, GDM criteria, control group criteria, family and personal risk of diabetes, gestational age, study design, control of risk factors during pregnancy (maternal age, gestational and pregestational body mass index (BMI), glucose, insulin, homeostatic model assessment (HOMA), glycosylated hemoglobin (A1cHb), maternal and fetal outcomes, follow-up period and patient loss assessment.

### 2.6. Evaluation of Quality and Risk of Bias

We used the Newcastle-Ottawa quality assessment scale (NOS) to assess the risk of bias [14]. Assessment was conducted by two reviewers independently who had content and methodological expertise (MMRR and CLT). The results were compared and conflicts resolved by agreement between the two reviewers, with input of a third reviewer as necessary. Studies that received a star in each domain were considered to be of high quality. The maximum score was 9, the minimum score 0. It was decided a priori that a score of 8 was reflective of high methodological quality (e.g., low risk of bias), a score of 6 or 7 indicated moderate quality and a score of 5 or less indicated low quality (e.g., high risk of bias).

### 2.7. Statistical Analysis

Means ± SD maternal leptin levels were extracted from primary-level studies to compare among GDM patients and controls. Since methodological heterogeneity was expected, mainly due to variations in laboratory determination methods (see protocol), the standardized mean difference (SMD) was chosen as effect size measure, estimated by Cohen’s d method with their corresponding 95% confidence intervals (CI). Data expressed as order statistics (i.e., medians with interquartile range and/or maximum-minimum values) were computed and transformed into means ± SD using the methods proposed by Luo et al. [15] and Wan et al. [16]. If it was desirable to combine two or more different means ± SD from subgroups into a single group, the method provided by Cochrane Handbook was followed [11]. When data were only expressed graphically the extraction was performed using Engauge-Digitizer 4.1 (open-source digitizing software developed by M. Mitchell). In the meta-analysis, SMDs with 95%CIs were pooled using the inverse-variance method under a random-effects model (based on the DerSimonian and Laird method), which accounts for the possibility that are different underlying results among study subpopulations (i.e., leptin variations among tissues, linked to geographical areas, or related to the inherent heterogeneity of the wide range of experimental methods). Forest plots were constructed to graphically represent the overall effect and for subsequent visual inspection analysis (*p* < 0.05 was considered significant). Statistical heterogeneity was evaluated applying the χ^2^-based Cochran’s Q test (given its low statistical power, *p* < 0.10 was considered significant) and quantified using Higgins I^2^ statistic (values of 50–75% were interpreted as moderate-to-high degree of inconsistency across the studies), which estimates what proportion of the variance in observed effects reflects variation in true effects, rather than sampling error [17,18]. Preplanned stratifications (by geographical area, trimester, determination technique, sample source, study design and risk of bias) and univariable meta-regression analyses (by age, gestational age, gestational and pregestational and gestational BMI, glycemia levels, insulin, HbA1c, HOMA, gestational age delivery, caesarian, newborn weight, and macrosomy) were conducted to identify potential sources of heterogeneity and to explore the potential variation of leptin levels on these subgroups [19]. For illustrative purposes, weighted bubble plots were also constructed to graphically represent the fitted meta-regression lines. Sensitivity analyses were additionally performed to test the reliability of our results, evaluating the influence of each individual study on the pooled estimations. For this purpose, the meta-analyses were repeated sequentially omitting one study each time (classic “leave-one-out” method). Finally, a small study effects analysis was performed through the assessment of funnel plots and the Egger regression test (*p* < 0.10 considered significant) [20,21]. Stata version 16.1 (Stata Corp, College Station, TX, USA) was employed for all tests, manually typing the commands syntax (PRG) [22].

## 3. Results

### 3.1. Results of the Literature Search

**Figure 1:**
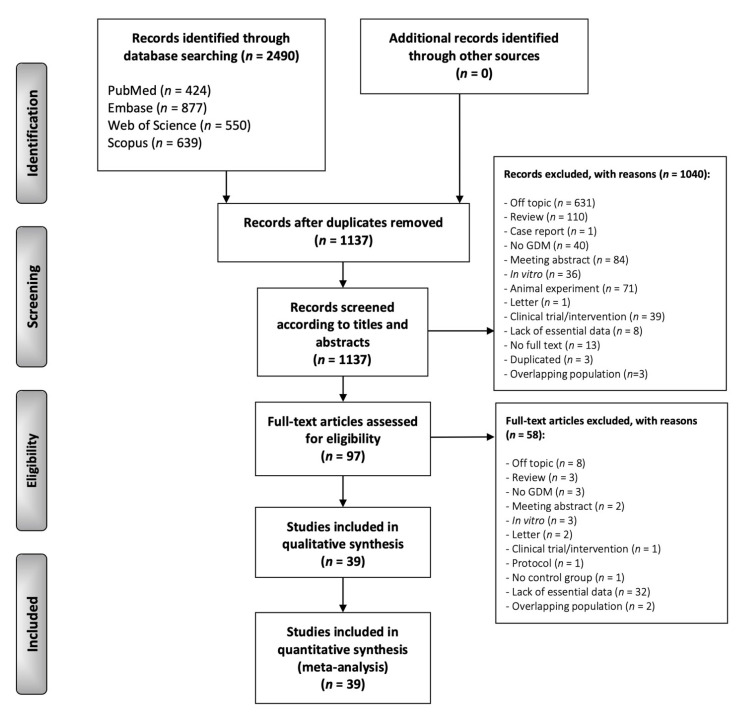
Flow diagram. Identification and selection process of relevant studies comparing leptin levels between GDM patients and controls.

The flow diagram (Figure 1) depicts the identification and selection process of studies. We retrieved a total of 2490 records published before 14 April 2021: 424 from MEDLINE/PubMed, 877 from Embase, 550 from the Web of Science, and 639 from Scopus. After eliminating duplicates, 1137 studies were considered potentially eligible (all the studies excluded and their exclusion criteria are listed in Figure 1). After screening their titles and abstracts, 97 were selected for full-text reading. After excluding studies that did not meet all eligibility criteria, 39 studies were finally included in the review for qualitative evaluation and quantitative meta-analysis.

### 3.2. Study Characteristics

Table 1 summarizes the characteristics of the 39 selected studies comparing the changes in circulating leptin levels on a total of 6101 patients (2255 GDM and 3846 control pregnant women) and Table S2 exhibits in more detail the variables gathered from each study. Source of samples were maternal blood serum in 10 studies, maternal blood plasma in 26 studies, and not specified in 3 studies. Leptin was quantified by ELISA in 32 studies and by RIA in 7 studies. Sample sizes ranged between 11 and 675 women. The studies were conducted in all continents except for Antarctica and South America and comprised the following geographical regions: 18 in Europe, 13 in Asia, 4 in North America, 1 in Central America, 2 in Oceania, and 1 in Africa.

### 3.3. Qualitative Evaluation

The qualitative analysis was conducted using the Newcastle–Ottawa Scale, which evaluates potential sources of bias in nine domains (Table 2):

In our revision, we only include studies in which the groups of diabetic patients are adequately selected and matched between conditions with their respective controls. Studies without a non-GDM comparator group were excluded. According to the overall Rob the studies were categorized 33.3% as low risk, 46.2% as moderate risk and 20.5% as high risk of potential bias. All studies showed a representativeness of the GDM and control patients (92.3% and 97.4% defined exactly the diagnostic criteria for GDM and controls, respectively), and 100% of studies displayed properly leptin quantification. The analysis revealed that the most frequent biases could be the inadequate description of maternal or fetal outcomes and failure to report on an appropriate follow-up period. In this regard, the risk of bias, with respect to the follow up and attrition rate, was elevated in 82.1% of the studies. It is worth highlighting the relevance of declare the lost to the follow-up which are essential data to evaluate any differences on obstetric and perinatal outcomes and on the subsequent follow-up and development of complications in both, the child and the mother.

### 3.4. Quantitative Evaluation (Meta-Analysis)

**Figure 2:**
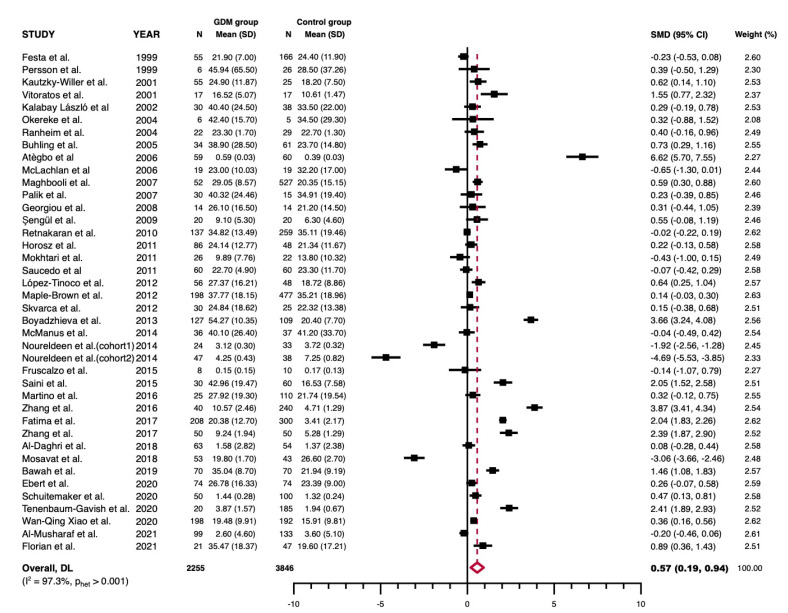
Forest plot. Forest plot graphically representing the meta-analysis evaluating the changes in circulating leptin levels between GDM patients and controls (random-effects model, inverse-variance weighting based on the DerSimonian and Laird method). Standardized mean difference (SMD) was chosen as effect size measure. An SMD > 0 suggests that leptin levels are higher in GDM. Diamond indicates the overall pooled SMDs with their corresponding 95% confidence intervals (CI).

*Meta-analysis on leptin levels in GDM.* Leptin levels were significantly higher in GDM participants than in controls (SMD = 0.57, 95%CI = 0.19 to 0.94; *p* < 0.001). Significant heterogeneity was observed (*p* < 0.001; I^2^ = 97.3%) (Figure 2, Table 3).

*Analysis of subgroups.* Several subgroups maintained the precedent significant association (some of them were identified as potential explanatory sources of heterogeneity and/or harbored a large effect size), by geographical area (Africa: SMD = 1.46, 95%CI = 1.08 to 1.83, *p* < 0.001; Europe: SMD = 0.92, 95%CI = 0.37 to 1.47, *p* = 0.001), by analysis technique (ELISA: SMD = 0.66, 95%CI = 0.23 to 1.10, *p* = 0.003), by sample source (plasma: SMD = 0.26, 95%CI = 0.01 to 0.52, *p* = 0.04; serum: SMD = 0.69, 95%CI = 0.16 to 1.21, *p* = 0.01), by study design (prospective studies: SMD = 0.87, 95%CI = 0.42 to 1.31; *p* < 0.001; loss of patients assessment: SMD = 1.92, 95%CI = 0.77 to 3.06, *p* = 0.001), and by risk of bias (low risk of bias/higher methodological quality: SMD = 1.31, 95%CI = 0.53 to 2.08, *p* = 0.001) (Table 3, Figures S1–S10).

*Univariable meta-regression analyses.* A positive significant relationship was found for the co-variate HOMA in the GDM group (*p* = 0.05), presenting the higher summary scores those primary-level studies showing higher positive SDMs for leptin levels. Significant differences were not found among the rest of study covariates (i.e., age of patients, gestational age, BMI, glycemia levels, insulin levels, HbA1c levels, and gestational complications) (Table 3, Figures S11–S22).

### 3.5. Quantitative Evaluation (Secondary Analyses)

*Small-study effects analysis.* The visual inspection analysis of the asymmetry of the funnel plot constructed (Figure S23) and the statistical test conducted for the same purpose (p_Egger_ = 0.765) confirmed the absence of “small-study” effects on the results of this meta-analysis. Therefore, the presence of biases, singularly publication bias, could be potentially ruled out.

*Sensitivity analysis.* The general results were stable, i.e., no substantial variations were observed after the sequential repetition of meta-analyses, omitting one study each time. This suggests that the combined estimations reported do not depend on the influence of a particular primary-level study (Figure S24).

## 4. Discussion

This meta-analysis which examined 39 studies and 6101 patients, showed that leptin levels were significantly higher in GDM participants than in controls (SMD = 0.57, 95%CI = 0.19 to 0.94; *p* < 0.001). These results are in line with Xu et al. [23] and Bao et al. who found that GDM patients had significantly higher serum leptin concentration than controls [7]. Sweeting et al. developed a first-trimester risk prediction model incorporating new maternal lipid and adipokine markers to accurately identify women at high risk of GDM, which incorporated new maternal lipid and adipokine markers to accurately identify women at high risk of GDM and observed higher levels of leptin in women who developed gestational diabetes, confirming its role as a predictor of GDM [24]. In the same way, Di Filippo et al. [25] also identified leptin as a promising diagnostic biomarker of GDM assessed against the oral glucose tolerance test (OGTT) from the second trimester to birth with a sensitivity and specificity >90% in adequate sample sizes (≥ 100). During pregnancy circulating leptin concentration is increased respect to non-pregnant women, reaching a peak around 28 gestational weeks and returning to pre-pregnancy levels postpartum. Leptin regulates the gonadotropin-releasing hormone and has several functions such as the promotion of embryo implantation, growth, development and fetal organogenesis. In trophoblastic cells, leptin induces the chorionic gonadotropin production, regulates placental growth, and stimulates the mitogenesis and amino acid uptake [8].

Our meta-analysis did not detect significant differences in leptin in the different trimesters of pregnancy, so there does not seem to be a more appropriate time than another for its determination. However, in order to establish strategies for early detection and selection of patients at higher risk, studies should be carried out to determine leptin levels in the early stages of pregnancy or even preconception. Nevertheless, our analysis detected higher leptin levels in the European studies, although most studies do not specify the ethnicity of the patients. This is an aspect of potential relevance that should be analyzed in future research.

In the meta-regression analyses, a significant positive relationship was found for the co-variate HOMA in the GDM group (*p* = 0.05), presenting the higher summary scores those primary-level studies showing higher positive SDMs for leptin levels. Significant differences were not found among the rest of study covariates. However, Xu et al. [23], assessing the effect of BMI on maternal leptin level by subgroup analysis, found that plasma leptin concentration remained significantly elevated in GDM patients compared to their BMI matched control subjects. In pregnancy, the placental production of leptin represents the major sources of higher levels of maternal circulating leptin and contributes to maternal fat mass gain which could further aggravate the insulin resistance associated with pregnancy and the onset of GDM. In fact, obese pregnant women have significantly elevated plasma leptin concentrations compared with non-obese pregnant women throughout pregnancy. It has been hypothesized as a mechanism leading to an accelerated fetal growth trajectory and macrosomia [3].

According to our qualitative evaluation -carried out using the Newcastle-Ottawa Scale (NOS)- all primary-level studies systematically reviewed were not conducted with the same rigor, although in general, most of them harbored low risk of potential bias across several domains. After applying a subgroup meta-analysis with sensitivity analysis purposes -i.e., to assess the influence of subsets of studies with lower risk of bias on the overall results-, we found that the studies presenting lower risk of bias significantly showed higher differences according to leptin levels between GDM subjects and controls (*p* = 0.001). This fact increases the quality of the evidence of the results reported in our meta-analysis, which could even be underestimated, due to the narrower and less robust differences reported by the studies harboring a higher risk of bias [26]. Therefore, future studies assessing the relationships between leptin levels and among GDM women should consider the potential biases and recommendations reported in this systematic review and meta-analysis, to improve and standardize future research.

To the best of our knowledge, this systematic review and meta-analysis, provides novel and more robust evidence to that previously published by Xu et al. [23] who evaluated twenty-seven studies only from PubMed/Medline, between 1966 and 2012 and restricted to English language. On the other hand, Bao et al. [7] who analyzed only eight prospective studies on leptin through 21 October 2014 from electronic search in only two databases, PubMed and EMBASE, and restricted to English language, without qualitative and risk of bias assessment. Based on these reasons, it seems appropriate to update our meta-analysis due to the time elapsed, designed and conducted under methodological criteria based on high standards and the highest quality of evidence to date (particularly 4 databases, more updated, no language limitation, larger sample size and more accurate results, assessment of the risk of bias and its influence on our meta-analytical results).

A potential limitation of this study should also be discussed, our meta-analysis revealed a considerable degree of heterogeneity. Heterogeneity is a common finding in meta-analyses dealing with biomarkers from serum and plasma measured and expressed as continuous variable [23]. Expectedly, as indicated in our study protocol, a random-effects model was a priori designed and applied in all meta-analyses to account for heterogeneity. When considering the uses and limitations of meta-analytical techniques, heterogeneity is considered as a major limitation. However, the other side of the coin is that one of the most key strengths of a meta-analysis is the ability to reveal patterns across study results, potentially explaining heterogeneity and identifying potential subpopulations (also known as sources of heterogeneity) [26]. In this sense, our meta-analysis may have identified differences among geographical regions, plasma and serum samples, variations in HOMA index, methodological aspects and risk of bias in primary level studies, among other factors, where more homogeneous subgroups were pooled that could constitute true sources of heterogeneity, potentially exerting an impact on leptin levels variations in GDM. Despite this potential above limitation, study strengths include our careful study design, an exhaustive literature search strategy, not limited by date or publication language filters; a robust qualitative analysis offering recommendations for the development and design of future studies on this topic; and a comprehensive meta-analytical approach, providing robust results (i.e., stable and reliable, as confirmed after sensitivity and small-study effects analyses, respectively). It should also be emphasized the large number of studies included, the comparable size of the control groups and the comparison of different countries.

## 5. Conclusions

Leptin emerges as a promising biomarker of GDM, but the heterogeneity of the methods used in the articles published so far may limit the external validity of the results and make it difficult to analyze the data properly. Due to short- and long-lasting health consequences of GDM, such as adverse perinatal-obstetric outcomes and increased risk of subsequent metabolic and cardiovascular disease in mother and child, and the lack of a widely accepted treatment or prevention strategy for GDM (except lifestyle intervention with diet and exercise, and insulin therapy), there is a need to discover early predictors of GDM risk that would allow intervention and prevention in high-risk women. It seems that high levels of leptin in the serum/plasma of pregnant women can be used as predictive markers in GDM. Future studies are needed to determine which biomolecules have the most potential to predict GDM and its complications.

## Figures and Tables

**Table 1:** Summarized characteristics of reviewed studies.

Total	39 studies
Year of publication	1999–2021
Number of patients	
Total	6101 patients
Cases with GDM	2255 patients
Controls	3846 patients
Sample size, range	11–675 patients
Leptin determination	
ELISA	32 studies
RIA	7 studies
Source of samples	
Maternal blood serum	10 studies
Maternal blood plasma	26 studies
Serum or plasma not specified	3 studies
Geographical region	
Europe	18 studies
Asia	13 studies
North America	4 studies
Central America	1 study
Oceania	2 studies
Africa	1 study

**Table 2:** Summary of risk of bias assessment using the specific tool Newcastle-Ottawa Quality Assessment Scale. Two reviewers who had content and methodological expertise independently assessed the risk of bias across the primary-level studies included in the present systematic review and meta-analysis, applying an adapted version of the Newcastle-Ottawa scale (NOS). The assessments were compared and conflicts resolved by consensus. The maximum score was 9, the minimum score 0. It was decided a priori that a score higher or equal to 8 was reflective of high methodological quality (e.g., low risk of bias), a score of 6 or 7 indicated moderate quality, and lower or equal to 5 indicated low quality (e.g., high risk of bias). Filled blue stars graphically represent that a star has been awarded (i.e., positive critical evaluation), and a white star graphically depict that no star has been awarded (i.e., negative critical evaluation).

Study	Selection		Control			Outcomes				Total Score	Overall RoB
	Selection GDM Patients	Selection Non-GDM Subjects	Family/ Personal GDM Risk Factors	Risk Factors during Pregnancy	Properly Leptin Quantification	Maternal Outcomes	Fetal Outcomes	Appropriate Follow-Up Period	Adequacy Follow-Up (Attrition)		
Festa et al., 1999	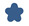	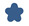	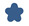	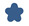	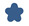	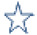	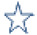	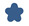	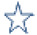	6	
Persson et al., 1999	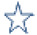	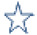	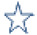	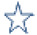	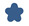	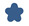	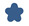	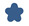	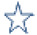	4	
Vitoratos et al., 2001	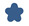	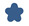	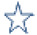	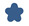	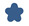	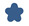	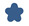	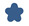	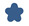	8	
Kautzky-Willer et al., 2001	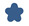	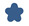	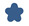	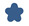	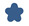	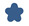	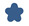	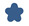	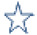	8	
Kalabay László et al., 2002	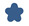	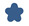	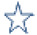	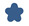	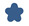	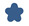	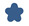	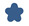	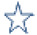	7	
Ranheim T et al., 2004	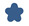	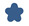	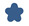	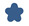	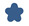	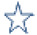	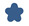	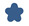	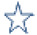	7	
Okereke NC et al., 2004	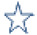	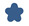	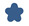	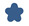	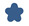	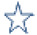	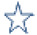	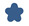	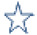	5	
Buhling KJ et al., 2005	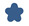	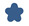	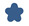	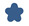	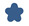	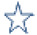	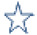	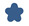	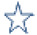	6	
McLachlan KA et al., 2006	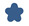	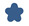	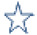	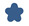	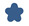	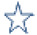	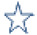	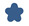	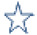	5	
Atègbo JM et al., 2006	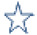	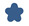	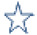	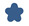	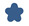	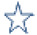	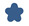	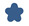	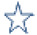	5	
Palik E et al., 2007	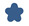	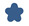	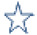	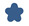	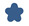	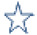	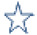	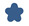	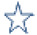	5	
Maghbooli Z et al., 2007	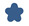	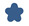	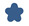	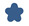	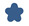	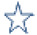	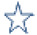	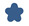	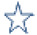	6	
Georgiou HM et al., 2008	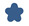	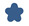	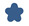	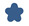	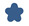	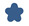	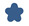	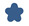	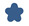	9	
Şengül et al., 2009	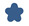	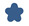	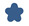	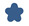	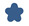	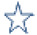	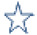	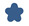	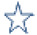	6	
Retnakaran R et al., 2010	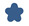	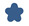	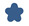	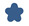	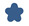	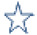	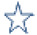	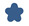	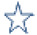	6	
Mokhtari M et al., 2011	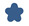	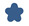	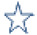	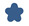	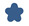	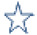	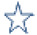	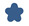	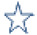	5	
Saucedo R et al., 2011	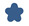	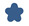	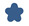	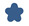	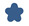	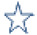	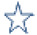	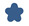	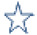	6	
Horosz et al., 2011	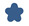	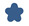	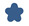	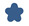	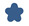	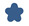	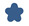	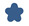	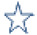	8	
López-Tinoco C et al., 2012	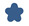	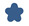	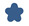	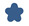	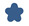	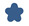	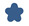	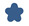	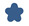	9	
Maple-Brown L et al., 2012	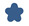	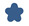	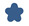	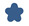	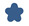	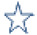	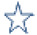	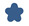	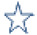	6	
Skvarca et al., 2012	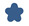	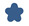	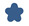	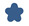	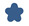	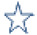	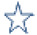	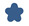	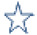	6	
Boyadzhieva M et al., 2013	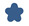	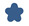	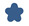	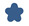	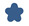	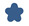	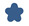	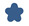	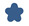	9	
McManus R et al., 2014	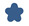	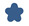	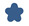	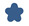	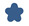	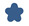	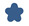	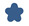	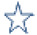	8	
Noureldeen AFH et al., 2014	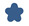	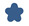	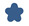	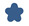	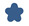	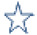	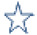	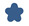	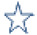	6	
Saini V et al., 2015	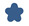	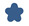	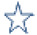	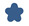	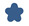	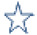	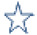	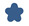	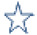	5	
Fruscalzo A et al., 2015	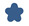	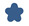	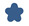	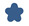	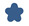	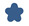	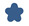	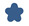	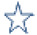	8	
Zhang Y et al., 2016	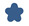	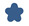	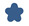	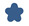	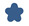	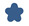	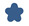	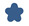	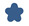	9	
Martino J et al., 2016	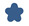	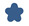	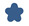	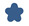	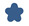	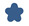	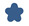	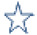	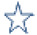	7	
Fatima SS et al., 2017	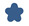	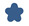	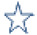	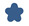	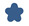	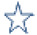	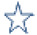	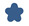	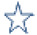	5	
Zhang Y et al., 2017	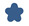	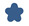	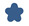	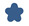	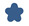	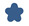	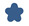	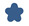	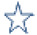	8	
Al-Daghri NM et al., 2018	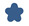	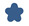	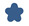	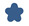	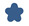	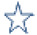	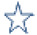	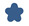	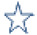	6	
Mosavat M et al., 2018	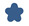	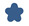	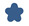	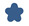	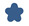	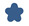	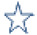	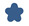	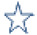	7	
Bawah et al., 2019	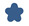	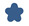	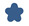	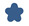	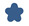	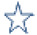	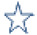	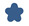	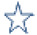	6	
Wan-Qing Xiao et al., 2020	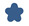	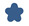	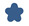	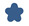	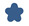	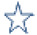	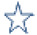	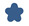	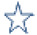	6	
Ebert T et al., 2020	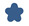	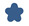	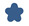	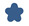	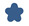	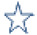	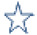	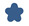	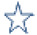	6	
Tenenbaum-Gavish et al., 2020	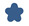	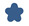	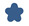	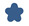	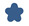	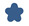	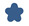	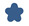	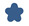	9	
Schuitemaker JHN et al., 2020	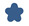	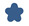	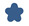	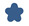	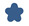	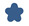	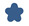	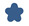	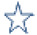	8	
Al-Musharaf S et al., 2021	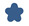	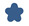	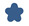	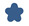	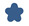	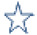	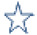	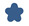	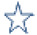	6	
Florian AR et al., 2021	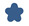	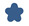	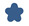	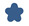	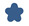	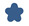	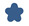	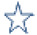	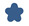	8	

Overall risk of bias (RoB): 8–9 low, 6–7 moderated and ≤5 high quality.

**Table 3:** Meta-analyses on circulating leptin levels in GDM.

					Pooled Data		Heterogeneity		
Meta-Analyses	No. of Studies	No. of Patients	Stat. Model	Wt	SMD (95%CI)	*p*-Value	*P_het_*	*I*^2^ (%)	Supplementary Material ^a^
All^b^	40	6101	REM	D-L	0.57 (0.19 to 0.94)	0.003	<0.001	97.3	Manuscript, Figure 2
Subgroup analysis by geographical area ^c^									Figure S1
Africa	1	140	──	──	1.46 (1.08 to 1.83)	<0.001	──	──	
Asia	14	2827	REM	D-L	0.31 (−0.52 to 1.14)	0.46	<0.001	98.5	
Central America	1	120	──	──	−0.07 (−0.42 to 0.29)	0.71	──	──	
Europe	18	1793	REM	D-L	0.92 (0.37 to 1.47)	0.001	<0.001	96.0	
North America	4	1155	REM	D-L	0.07 (−0.05 to 0.20)	0.26	0.65	0.0	
Oceania	2	66	REM	D-L	−0.19 (−1.12 to 0.75)	0.69	0.06	71.7	
Subgroup analysis by trimester ^c^									Figure S2
First	6	766	REM	D-L	0.81 (−0.02 to 1.64)	0.06	<0.001	95.2	
Second	11	2506	REM	D-L	0.44 (−0.53 to 1.41)	0.37	<0.001	98.7	
Third	21	2626	REM	D-L	0.55 (0.12 to 0.98)	0.13	<0.001	95.7	
Not reported	2	203	REM	D-L	0.58 (0.02 to 1.15)	0.04	0.10	62.7	
Subgroup analysis by trimester ^c^									Figure S3
First	6	766	REM	D-L	0.81 (−0.02 to 1.64)	0.06	<0.001	95.2	
Other	32	5132	REM	D-L	0.52 (0.08 to 0.96)	0.21	<0.001	97.7	
Not reported	2	203	REM	D-L	0.58 (0.02 to 1.15)	0.04	0.10	62.7	
Subgroup analysis by trimester ^c^									Figure S4
Second	11	2506	REM	D-L	0.44 (−0.53 to 1.41)	0.37	<0.001	98.7	
Other	27	3392	REM	D-L	0.60 (0.23 to 0.98)	<0.001	<0.001	95.5	
Not reported	2	203	REM	D-L	0.58 (0.02 to 1.15)	0.04	0.10	62.7	
Subgroup analysis by trimester ^c^									Figure S5
Third	21	2626	REM	D-L	0.55 (0.12 to 0.98)	0.13	<0.001	95.7	
Other	17	3272	REM	D-L	0.57 (−0.11 to 1.26)	0.10	<0.001	98.2	
Not reported	2	203	REM	D-L	0.58 (0.02 to 1.15)	0.04	0.10	62.7	
Subgroup analysis by analysis technique ^c^									Figure S6
ELISA	33	5548	REM	D-L	0.66 (0.23 to 1.10)	0.003	<0.001	97.7	
RIA	7	553	REM	D-L	0.07 (−0.26 to 0.40)	0.66	0.02	61.9	
Subgroup analysis by sample source ^c^									Figure S7
Plasma	10	1112	REM	D-L	0.26 (0.01 to 0.52)	0.04	<0.001	70.3	
Serum	27	4731	REM	D-L	0.69 (0.16 to 1.21)	0.01	<0.001	98.0	
Not reported	3	258	REM	D-L	0.52 (−0.89 to 1.93)	0.47	<0.001	96.1	
Subgroup analysis by prospective vs. retrospective design ^c^									Figure S8
Prospective	30	4812	REM	D-L	0.87 (0.42 to 1.31)	<0.001	<0.001	97.6	
Retrospective	10	1289	REM	D-L	−0.32 (−0.98 to 0.34)	0.34	<0.001	95.2	
Subgroup analysis by loss of patient assessment ^c^									Figure S9
No	33	5146	REM	D-L	0.28 (−0.08 to 0.63)	0.12	<0.001	96.6	
Yes	7	955	REM	D-L	1.92 (0.77 to 3.06)	0.001	<0.001	97.1	
Subgroup analysis by RoB ^c^									Figure S10
High-moderate RoB	27	4591	REM	D-L	0.21 (−0.20 to 0.61)	0.32	<0.001	97.0	
Low RoB	13	1510	REM	D-L	1.31 (0.53 to 2.08)	0.001	<0.001	97.0	
Univariable meta-regression ^d^									
Gestational age in GDM (weeks)	38	5898	random-effects meta-regression		Coef = 0.021 (−0.056 to 0.098)	0.60 ± 0.005 ^e^	het_explained_ = −2.09% ^f^		Figure S11
Age in GDM (years)	37	5860	random-effects meta-regression		Coef = −0.118 (−0.329 to 0.093)	0.26 ± 0.004 ^e^	het_explained_ = 0.77% ^f^		Figure S12
Pregestational BMI in GDM (summary index score)	19	3382	random-effects meta-regression		Coef = −0.216 (−0.510 to 0.077)	0.14 ± 0.004 ^e^	het_explained_ = 7.53% ^f^		Figure S13
Gestational BMI in GDM (summary index score)	32	4370	random-effects meta-regression		Coef = −0.159 (−0.365 to 0.048)	0.10 ± 0.003 ^e^	het_explained_ = 5.04% ^f^		Figure S14
Glycemia levels in GDM (mmol/l)	24	3438	random-effects meta-regression		Coef = −0.557 (−0.258 to 1.37)	0.15 ± 0.004 ^e^	het_explained_ = 4.27% ^f^		Figure S15
Insulin in GDM (pmol/l)	18	3935	random-effects meta-regression		Coef = 0.004 (−0.007 to 0.014)	0.38 ± 0.005 ^e^	het_explained_ = −2.82% ^f^		Figure S16
HbA1c in GDM (%)	14	1796	random-effects meta-regression		Coef = 0.803 (−1.930 to 3.535)	0.52 ± 0.005 ^e^	het_explained_ = −4.98% ^f^		Figure S17
HOMA in GDM (summary index score)	13	2306	random-effects meta-regression		Coef = 0.430 (−0.008 to 0.868)	0.05 ± 0.002 ^e^	het_explained_ = 23.88% ^f^		Figure S18
Gestational age delivery in GDM (weeks)	15	1638	random-effects meta-regression		Coef = −0.097 (−1.521 to 1.329)	0.89 ± 0.003 ^e^	het_explained_ = −7.60% ^f^		Figure S19
Caesarian in GDM (%)	7	892	random-effects meta-regression		Coef = −0.008 (−0.113 to 0.096)	0.79 ± 0.004 ^e^	het_explained_ = −19.47% ^f^		Figure S20
Newborn weight in GDM (gr)	15	1684	random-effects meta-regression		Coef = 0.003 (−0.001 to 0.006)	0.12 ± 0.003 ^e^	het_explained_ = 12.49% ^f^		Figure S21
Macrosomy in GDM (%)	5	873	random-effects meta-regression		Coef = 0.060 (−0.071 to 0.192)	0.31 ± 0.005 ^e^	het_explained_ = 21.92% ^f^		Figure S22

Abbreviations: Stat., statistical; Wt, method of weighting; SMD, standardized mean difference; CI, confidence intervals; REM, random-effects model; D-L, DerSimonian and Laird method; GDM, gestational diabetes mellitus; NR, not reported. ^a^ More information in the appendix. ^b^ Meta-analysis. ^c^ Subgroup meta-analysis. ^d^ Effect of study covariates on circulating leptin levels among patients with GDM compared with controls, estimated using SMD as effect size measure. A meta-regression coefficient >0 indicates a greater impact of covariates on effect size. ^e^ *p*-value ± standard error after 10,000 permutations based on Monte Carlo simulation. ^f^ Proportion of between-study variance explained (adjusted R^2^ statistic), expressed as percentage, using the residual maximum likelihood (REML) method. A negative proportion reflects no heterogeneity explained.

## Data Availability

The data that support the findings of this study are available in the supplementary material of this article.

## Supplementary Materials

The following supporting information can be downloaded at: https://www.mdpi.com/article/10.3390/jcm11092433/s1, Table S1. Search strategy for each database, number of results, and execution date; Full-text articles excluded (n = 58) list; Table S2. Characteristics of analyzed studies (n = 39); Figure S1. Forest plot graphically representing the subgroup meta-analysis evaluating the changes in circulating leptin levels between GDM patients and healthy control women, stratified by geographical area; Figure S2. Forest plot graphically representing the subgroup meta-analysis evaluating the changes in circulating leptin levels between GDM patients and healthy control women, stratified by trimester (first vs second vs third); Figure S3. Forest plot graphically representing the subgroup meta-analysis evaluating the changes in circulating leptin levels between GDM patients and healthy control women, stratified by trimester (first vs other); Figure S4. Forest plot graphically representing the subgroup meta-analysis evaluating the changes in circulating leptin levels between GDM patients and healthy control women, stratified by trimester (second vs other); Figure S5. Forest plot graphically representing the subgroup meta-analysis evaluating the changes in circulating leptin levels between GDM patients and healthy control women, stratified by trimester (third vs other); Figure S6. Forest plot graphically representing the subgroup meta-analysis evaluating the changes in circulating leptin levels between GDM patients and healthy control women, stratified by type of analysis (ELISA vs RIA); Figure S7. Forest plot graphically representing the subgroup meta-analysis evaluating the changes in circulating leptin levels between GDM patients and healthy control women, stratified by type of analysis (plasma vs serum); Figure S8. Forest plot graphically representing the subgroup meta-analysis evaluating the changes in circulating leptin levels between GDM patients and healthy control women, stratified by study design (prospective vs. retrospective); Figure S9. Forest plot graphically representing the subgroup meta-analysis evaluating the changes in circulating leptin levels between GDM patients and healthy control women, stratified by the assessment of the loss of patients; Figure S10. Forest plot graphically representing the subgroup meta-analysis evaluating the changes in circulating leptin levels between GDM patients and healthy control women, stratified by risk of bias (low vs. moderate-high); Figure S11. Bubble plot graphically representing the univariable meta-regression analysis of the potential effect of gestational age (weeks) on the circulating leptin levels among patients with GDM compared with healthy control women; Figure S12. Bubble plot graphically representing the univariable meta-regression analysis of the potential effect of age (years) on the circulating leptin levels among patients with GDM compared with healthy control women; Figure S13. Bubble plot graphically representing the univariable meta-regression analysis of the potential effect of pregestational BMI (summary index score) on the circulating leptin levels among patients with GDM compared with healthy control women; Figure S14. Bubble plot graphically representing the univariable meta-regression analysis of the potential effect of gestational BMI (summary index score) on the circulating leptin levels among patients with GDM compared with healthy control women; Figure S15. Bubble plot graphically representing the univariable meta-regression analysis of the potential effect of glycemia levels (mmol/l) on the circulating leptin levels among patients with GDM compared with healthy control women; Figure S16. Bubble plot graphically representing the univariable meta-regression analysis of the potential effect of insulin (pmol/l) on the circulating leptin levels among patients with GDM compared with healthy control women; Figure S17. Bubble plot graphically representing the univariable meta-regression analysis of the potential effect of HbA1c (mg/dl) on the circulating leptin levels among patients with GDM compared with healthy control women; Figure S18. Bubble plot graphically representing the univariable meta-regression analysis of the potential effect of HOMA (summary index score) on the circulating leptin levels among patients with GDM compared with healthy control women; Figure S19. Bubble plot graphically representing the univariable meta-regression analysis of the potential effect of gestational age delivery (weeks) on the circulating leptin levels among patients with GDM compared with healthy control women; Figure S20. Bubble plot graphically representing the univariable meta-regression analysis of the potential effect of caesarian (%) on the circulating leptin levels among patients with GDM compared with healthy control women; Figure S21. Bubble plot graphically representing the univariable meta-regression analysis of the potential effect of newborn weight (gr) on the circulating leptin levels among patients with GDM compared with healthy control women; Figure S22. Bubble plot graphically representing the univariable meta-regression analysis of the potential effect of macrosomy (%) on the circulating leptin levels among patients with GDM compared with healthy control women; Figure S23. Funnel plot. Funnel plots of the estimated circulating leptin levels comparing GDM patients and healthy control women, expressed as standardized mean difference (SMD) against its standard error; Figure S24. Interval plot graphically representing the sensitivity analysis (“leave-one-out” method) of the studies pooled in the meta-analysis evaluating the changes in circulating leptin levels between GDM patients and healthy control women.
