# Supplementary material for: Significance of Serum-Plasma Leptin Profile during Pregnancy in Gestational Diabetes Mellitus: A Systematic Review and Meta-Analysis
Source: J Clin Med. 2022 Apr 26;11(9):2433. doi: 10.3390/jcm11092433 (PMC9102207; doi:10.3390/jcm11092433)
Supplement: Supplementary file 1 [file jcm-11-02433-s001.zip › jcm-1682032-supplementary.pdf]

Supplementary materials to the manuscript

Significance of serum-plasma leptin profile during pregnancy in gestational diabetes mellitus. A systematic review and meta-analysis

MM Roca-Rodríguez (1), P Ramos-García (2), C López-Tinoco (1), M Aguilar-Diosdado (1).

(1) Endocrinology and Nutrition Department, Cadiz Biomedical Research and Innovation Institute (INiBICA), Puerta del Mar University Hospital, Cadiz, Spain

(2) Oral Medicine Department, School of Dentistry, University of Granada, Granada, Spain.

Corresponding Authors: MM Roca-Rodríguez and P Ramos-García

1. Search strategy

Table S1. Search strategy for each database, number of results, and execution date.

Database	Query	Results	Upper date limit
PubMed	(“Diabetes, Gestational”[MeSH Terms] OR “Gestational diabetes”[All Fields] OR “Pregnancy in Diabetics”[MeSH Terms] OR “pregnancy diabetes mellitus”[All Fields] OR “GDM”[All Fields]) AND ("leptin"[MeSH Terms] OR "leptin"[All Fields])	424	April, 2021
Embase	('pregnancy diabetes mellitus'/exp OR 'pregnancy diabetes mellitus' OR 'Gestational diabetes' OR 'GDM') AND ('leptin'/exp OR 'leptin')	877	April, 2021
Web of Science	TS=("Gestational diabetes" OR "pregnancy diabetes mellitus" OR "GDM") AND TS=("leptin")	550	April, 2021
Scopus	TITLE-ABS-KEY((“Gestational diabetes” OR “pregnancy diabetes mellitus” OR “GDM”) AND “leptin”)	639	April, 2021
Total			2490

2. Full-text articles excluded (n=58)

Gross GA, Solenberger T, Philpott T, Holcomb WL Jr, Landt M. Plasma leptin concentrations in newborns of diabetic and nondiabetic mothers. *Am J Perinatol*. 1998;15(4):243-247. doi:10.1055/s-2007-993935

Lack of essential data

Lepercq J, Cauzac M, Lahlou N, et al. Overexpression of placental leptin in diabetic pregnancy: a critical role for insulin. *Diabetes*. 1998;47(5):847-850. doi:10.2337/diabetes.47.5.847

Lack of essential data

Sattar N, Greer IA, Pirwani I, Gibson J, Wallace AM. Leptin levels in pregnancy: marker for fat accumulation and mobilization?. *Acta Obstet Gynecol Scand*. 1998;77(3):278-283.

Lack of essential data

Lewandowski K, Horn R, O'Callaghan CJ, et al. Free leptin, bound leptin, and soluble leptin receptor in normal and diabetic pregnancies. *J Clin Endocrinol Metab*. 1999;84(1):300-306. doi:10.1210/jcem.84.1.5401

No GDM

Ng PC, Lam CW, Lee CH, et al. Leptin and metabolic hormones in infants of diabetic mothers. *Arch Dis Child Fetal Neonatal Ed*. 2000;83(3):F193-F197. doi:10.1136/fn.83.3.f193

Off Topic

Linnemann K, Malek A, Schneider H, Fusch C. Physiological and pathological regulation of feto/placento/maternal leptin expression. *Biochem Soc Trans*. 2001;29(Pt 2):86-90. doi:10.1042/0300-5127:0290086

In vitro

Simmons D, Breier BH. Fetal overnutrition in polynesian pregnancies and in gestational diabetes may lead to dysregulation of the adipoinsular axis in offspring. *Diabetes Care*. 2002;25(9):1539-1544. doi:10.2337/diacare.25.9.1539

Lack of essential data

Henson MC, Castracane VD. Leptin: roles and regulation in primate pregnancy. Semin Reprod Med. 2002;20(2):113-122. doi:10.1055/s-2002-32502

Review

Qiu C, Williams MA, Vadachkoria S, Frederick IO, Luthy DA. Increased maternal plasma leptin in early pregnancy and risk of gestational diabetes mellitus. *Obstet Gynecol*. 2004;103(3):519-525. doi:10.1097/01.AOG.0000113621.53602.7a

Lack of essential data

Krzyzanowska K, Krugluger W, Mittermayer F, et al. Increased visfatin concentrations in women with gestational diabetes mellitus. *Clin Sci (Lond)*. 2006;110(5):605-609. doi:10.1042/CS20050363

Lack of essential data

Álvarez Ballano D, Gracia Ruiz ML, Barragán Angulo A, et al. Leptina e insulinoterapia en la diabetes gestacional. Endocrinol nutr. 2006;53(10):582-586

Lack of essential data

Madarász E, Tabák AG, Speer G, Lakatos P, Kerényi Z, Tamás G. Abnormal glucose tolerance is associated with diminished postload change in leptin levels in women. *Diabetes Metab Res Rev*. 2009;25(7):632-638. doi:10.1002/dmrr.1001

Off topic

Kralisch S, Stepan H, Kratzsch J, et al. Serum levels of adipocyte fatty acid binding protein are increased in gestational diabetes mellitus. *Eur J Endocrinol*. 2009;160(1):33-38. doi:10.1530/EJE-08-0540

Lack of essential data

Gibson W, Liu J, Gaylinn B, et al. Effects of glucose and insulin on acyl ghrelin and desacyl ghrelin, leptin, and adiponectin in pregnant women with diabetes. *Metabolism*. 2010;59(6):841-847. doi:10.1016/j.metabol.2009.09.033

No GDM

McIntyre HD, Chang AM, Callaway LK, et al. Hormonal and metabolic factors associated with variations in insulin sensitivity in human pregnancy. *Diabetes Care*. 2010;33(2):356-360. doi:10.2337/dc09-1196

Lack of essential data

Retnakaran R, Qi Y, Connelly PW, Sermer M, Hanley AJ, Zinman B. Risk of early progression to prediabetes or diabetes in women with recent gestational dysglycaemia but normal glucose tolerance at 3-month postpartum. *Clin Endocrinol (Oxf)*. 2010;73(4):476-483. doi:10.1111/j.1365-2265.2010.03834.x

Overlapping population

Yilmaz O, Kucuk M, Ilgin A, Dagdelen M. Assessment of insulin sensitivity/resistance and their relations with leptin concentrations and anthropometric measures in a pregnant population with and without gestational diabetes mellitus. *J Diabetes Complications*. 2010;24(2):109-114. doi:10.1016/j.jdiacomp.2009.01.006

Lack of essential data

Fattah C, Barry S, O'connor N, Farah N, Stuart B, Turner MJ. Maternal leptin and body composition in the first trimester of pregnancy. *Gynecol Endocrinol*. 2011;27(4):263-266. doi:10.3109/09513590.2010.491167

Off topic

Soheilykhah S, Mojibian M, Rahimi-Saghand S, Rashidi M, Hadinedoushan H. Maternal serum leptin concentration in gestational diabetes. *Taiwan J Obstet Gynecol*. 2011;50(2):149-153. doi:10.1016/j.tjog.2011.01.034

Lack of essential data

Stuebe AM, Mantzoros C, Kleinman K, et al. Duration of lactation and maternal adipokines at 3 years postpartum. *Diabetes*. 2011;60(4):1277-1285. doi:10.2337/db10-0637

Off topic

Olmos PR, Borzone GR, Olmos RI, et al. Gestational diabetes and pre-pregnancy overweight: possible factors involved in newborn macrosomia. *J Obstet Gynaecol Res*. 2012;38(1):208-214. doi:10.1111/j.1447-0756.2011.01681.x

Off topic

Mendieta Zerón H, García Solorio VJ, Nava Díaz PM, et al. Hyperleptinemia as a prognostic factor for preeclampsia: a cohort study. *Acta Medica (Hradec Kralove)*. 2012;55(4):165-171. doi:10.14712/18059694.2015.41

Lack of essential data

Retnakaran R, Ye C, Hanley AJ, et al. Effect of maternal weight, adipokines, glucose intolerance and lipids on infant birth weight among women without gestational diabetes mellitus. *CMAJ*. 2012;184(12):1353-1360. doi:10.1503/cmaj.111154

Lack of essential data

Desai M, Beall M, Ross MG. Developmental origins of obesity: programmed adipogenesis. *Curr Diab Rep*. 2013;13(1):27-33. doi:10.1007/s11892-012-0344-x

Review

Disse E, Graeppi-Dulac J, Joncour-Mills G, Dupuis O, Thivolet C. Heterogeneity of pregnancy outcomes and risk of LGA neonates in Caucasian females according to IADPSG criteria for gestational diabetes mellitus. Diabetes Metab. 2013;39(2):132-138. doi:10.1016/j.diabet.2012.09.006

Lack of essential data

Park S, Kim MY, Baik SH, et al. Gestational diabetes is associated with high energy and saturated fat intakes and with low plasma visfatin and adiponectin levels independent of prepregnancy BMI. *Eur J Clin Nutr*. 2013;67(2):196-201. doi:10.1038/ejcn.2012.207

Lack of essential data

Retnakaran R, Ye C, Hanley A, et al. Effect of maternal gestational diabetes on the cardiovascular risk factor profile of infants at 1 year of age. *Nutr Metab Cardiovasc Dis*. 2013;23(12):1175-1181. doi:10.1016/j.numecd.2013.03.009

Overlapping population

Kramer CK, Hamilton JK, Ye C, et al. Antepartum determinants of rapid early-life weight gain in term infants born to women with and without gestational diabetes. *Clin Endocrinol (Oxf)*. 2014;81(3):387-394. doi:10.1111/cen.12437

Lack of essential data

Soltani Yacine S, Mehaoudi Rym Ikram M, Oussekhri Hanane O, et al. Screening and diagnosis of gestational diabetes: Impact of age, BMI, and adipokines. Obesity Reviews 2014; 15: 125-125. doi.org/10.1111/obr.12150

Meeting abstract

Zhao YN, Li Q, Li YC. Effects of body mass index and body fat percentage on gestational complications and outcomes. *J Obstet Gynaecol Res*. 2014;40(3):705-710. doi:10.1111/jog.12240

Lack of essential data

Dos Santos E, Duval F, Vialard F, Dieudonné MN. The roles of leptin and adiponectin at the fetal-maternal interface in humans. *Horm Mol Biol Clin Investig*. 2015;24(1):47-63. doi:10.1515/hmbci-2015-0031

In vitro

Honnorat D, Disse E, Millot L, et al. Are third-trimester adipokines associated with higher metabolic risk among women with gestational diabetes?. *Diabetes Metab*. 2015;41(5):393-400. doi:10.1016/j.diabet.2015.03.003

Lack of essential data

Pala HG, Ozalp Y, Yener AS, Gerceklioglu G, Uysal S, Onvural A. Adiponectin levels in gestational diabetes mellitus and in pregnant women without glucose intolerance. *Adv Clin Exp Med*. 2015;24(1):85-92. doi:10.17219/acem/38141

Off topic

Reynolds CM, Gray C, Li M, Segovia SA, Vickers MH. Early Life Nutrition and Energy Balance Disorders in Offspring in Later Life. *Nutrients*. 2015;7(9):8090-8111. Published 2015 Sep 21. doi:10.3390/nu7095384

Review

Sommer C, Jenum AK, Waage CW, Mørkrid K, Sletner L, Birkeland KI. Ethnic differences in BMI, subcutaneous fat, and serum leptin levels during and after pregnancy and risk of gestational diabetes. *Eur J Endocrinol*. 2015;172(6):649-656. doi:10.1530/EJE-15-0060

Lack of essential data

Garcia-Flores J, Cruceyra M, Cañamares M, et al. Weight-related and analytical maternal factors in gestational diabetes to predict birth weight and cord markers of diabetic fetopathy. *Gynecol Endocrinol*. 2016;32(7):548-552. doi:10.3109/09513590.2016.1138461

Lack of essential data

Jeon EJ, Hong SY, Lee JH. Adipokines and Insulin Resistance According to Characteristics of Pregnant Women with Gestational Diabetes Mellitus. *Diabetes Metab J*. 2017;41(6):457-465. doi:10.4093/dmj.2017.41.6.457

Lack of essential data

Beyazıt F, Ünsal MA. Obesity and insulin resistance are significant predictors of serum leptin levels. *J Turk Ger Gynecol Assoc*. 2017;18(3):158-159. doi:10.4274/jtgga.2017.0027

Letter

Fried RL, Mayol NL, McDade TW, Kuzawa CW. Maternal metabolic adaptations to pregnancy among young women in Cebu, Philippines. *Am J Hum Biol*. 2017;29(5):10.1002/ajhb.23011. doi:10.1002/ajhb.23011

No GDM

Guelfi KJ, Ong MJ, Li S, et al. Maternal circulating adipokine profile and insulin resistance in women at high risk of developing gestational diabetes mellitus. *Metabolism*. 2017;75:54-60. doi:10.1016/j.metabol.2017.08.003

Clinical/trial intervention

Perichart-Perera O, Muñoz-Manrique C, Reyes-López A, Tolentino-Dolores M, Espino Y Sosa S, Ramírez-González MC. Metabolic markers during pregnancy and their association with maternal and newborn weight status. *PLoS One*. 2017;12(7):e0180874. Published 2017 Jul 27. doi:10.1371/journal.pone.0180874

Off topic

Thagaard IN, Krebs L, Holm JC, Lange T, Larsen T, Christiansen M. Adiponectin and leptin as first trimester markers for gestational diabetes mellitus: a cohort study. *Clin Chem Lab Med*. 2017;55(11):1805-1812. doi:10.1515/cclm-2017-0427

Lack of essential data

Shang M, Dong X, Hou L. Correlation of adipokines and markers of oxidative stress in women with gestational diabetes mellitus and their newborns. J Obstet Gynaecol Res. 2018;44(4):637-646. doi:10.1111/jog.13586

Lack of essential data

Bugatto F, Quintero-Prado R, Visiedo FM, et al. The Influence of Lipid and Proinflammatory Status on Maternal Uterine Blood Flow in Women With Late Onset Gestational Diabetes. *Reprod Sci*. 2018;25(6):837-843. doi:10.1177/1933719117698576

Lack of essential data

Mohamad M, Loy SL, Lim PY, Wang Y, Soo KL, Mohamed HJJ. Maternal Serum and Breast Milk Adiponectin: The Association with Infant Adiposity Development. *Int J Environ Res Public Health*. 2018;15(6):1250. Published 2018 Jun 12. doi:10.3390/ijerph15061250

Lack of essential data

León-Reyes G, Guzmán-Grenfell AM, Medina-Navarro R, et al. Is gestational diabetes mellitus in obese women predicted by oxidative damage in red blood cells?. *Gynecol Endocrinol*. 2018;34(11):995-1000. doi:10.1080/09513590.2018.1473360

In vitro

Ryu OH. Adipokines and Insulin Resistance According to Characteristics of Pregnant Women with Gestational Diabetes Mellitus (Diabetes Metab J 2017;41:457-65). *Diabetes Metab J*. 2018;42(1):87-89. doi:10.4093/dmj.2018.42.1.87

Letter

Correa PJ, Venegas P, Palmeiro Y, et al. First trimester prediction of gestational diabetes mellitus using plasma biomarkers: a case-control study. *J Perinat Med*. 2019;47(2):161-168. doi:10.1515/jpm-2018-0120

Lack of essential data

Hinkle SN, Rawal S, Liu D, Chen J, Tsai MY, Zhang C. Maternal adipokines longitudinally measured across pregnancy and their associations with neonatal size, length, and adiposity. *Int J Obes (Lond)*. 2019;43(7):1422-1434. doi:10.1038/s41366-018-0255-2

Lack of essential data

Sweeting AN, Wong J, Appelblom H, et al. A Novel Early Pregnancy Risk Prediction Model for Gestational Diabetes Mellitus. *Fetal Diagn Ther*. 2019;45(2):76-84. doi:10.1159/000486853

Lack of essential data

Aviram A, Shtaif B, Gat-Yablonski G, Yogev Y. The association between adipocytokines and glycemic control in women with gestational diabetes mellitus. *J Matern Fetal Neonatal Med*. 2020;33(2):177-183. doi:10.1080/14767058.2018.1487944

Lack of essential data

O'Malley EG, Reynolds CME, Killalea A, O'Kelly R, Sheehan SR, Turner MJ. The use of biomarkers at the end of the second trimester to predict Gestational Diabetes Mellitus. Eur J Obstet Gynecol Reprod Biol. 2020;250:101-106. doi:10.1016/j.ejogrb.2020.04.064

No control group

Waters TP, Kim SY, Sharma AJ, et al. Longitudinal changes in glucose metabolism in women with gestational diabetes, from late pregnancy to the postpartum period. *Diabetologia*. 2020;63(2):385-394. doi:10.1007/s00125-019-05051-0

Lack of essential data

Zhang YZ, Zhou L, Tian L, et al. A mid-pregnancy risk prediction model for gestational diabetes mellitus based on the maternal status in combination with ultrasound and serological findings. *Exp Ther Med*. 2020;20(1):293-300. doi:10.3892/etm.2020.8690

Off topic

Francis EC, Li M, Hinkle S, et al. A longitudinal study of a panel of adipokines and gestational diabetes risk. *Diabetes* 2019; 68 (supplement_1): 1517. doi.org/10.2337/db19-1517-p

Meeting abstract

Tilva, H and Tayade, S. Serum Leptin levels, body mass index and its correlation with maternofetal outcome in gestational diabetes mellitus. European Journal of Molecular and Clinical Medicine. 2020; 7 (7): 2040-2048.

Protocol

Johnson AW, Snegovskikh D, Parikh L, DeAguiar RB, Han CS, Hwang JJ. Characterizing the Effects of Diabetes and Obesity on Insulin and Leptin Levels amongst Pregnant Women. *Am J Perinatol*. 2020;37(11):1094-1101. doi:10.1055/s-0040-1702988

Lack of essential data

Kapustin RV, Chepanov SV, Babakov VN, et al. Maternal serum leptin, adiponectin, resistin and monocyte chemoattractant protein-1 levels in different types of diabetes mellitus. *Eur J Obstet Gynecol Reprod Biol*. 2020;254:284-291. doi:10.1016/j.ejogrb.2020.09.050

Lack of essential data

3. Table S2. Characteristics of analyzed studies (n=39).

Study	Year	Country	Continent	No. of patients with GDM	M(+/-DE) Leptin GDM	No. of Patients controls	M(+/-DE) Leptin controls	Technique	Units	Source of data	Sample source	Study Design	prospective/retrospective	M (+/-DE) Age GDM	M (+/-DE) Age controls	M (+/-DE) BMI GDM	M (+/-DE) BMI controls	M F
Festa et al.	1999	Austria	Europe	55	21,9 ± 7	166	24,4 ± 11,9	RIA	ng/ml	mean-sd	Plasma	case-control	Prospective	29,4 ± 5,9	26,4 ± 5,2	28,8 ± 4,7	25,9 ± 4,3	4
Persson et al.	1999	Sweden	Europe	6	45,94 ± 65,5	26	28,5 ± 37,26	RIA	ng/ml	median-iqr	Plasma	case-control	prospective	N/A	N/A	N/A	N/A	N/
Kautzky-Willer et al.	2001	Austria	Europe	55	24,9 ± 11,87	25	18,2 ± 7,5	RIA	ng/ml	mean-sem	Plasma	case-control	prospective	30,9 ± 0,9	29,6 ± 1,9	28 ± 0,9	28,1 ± 0,8	4,
Vitoratos et al.	2001	Greece	Europe	17	16,52 ± 5,07	17	10,61 ± 1,47	ELISA	ng/ml	mean-sd	Serum	case-control	prospective	34,2 ± 3,1	33 ± 2,3	36,74 ± 6,52	34,86 ± 5,91	N/
Kalabay László et al	2002	Hungary	Europe	30	40,4 ± 24,5	38	33,5 ± 22	ELISA	ng/ml	mean-sd	Serum	case-control	retrospective	28 ± 2,8	27,8 ± 2,8	33,4 ± 6,4	25,8 ± 2,7	4,
Okereke et al.	2004	USA	North America	6	42,4 ± 15,7	5	34,5 ± 29,3	RIA	ng/ml	mean-sd	Serum	case-control	prospective	29,9 ± 4,1	31,6 ± 3,4	N/A	N/A	N/
Ranheim et al.	2004	Norway	Europe	22	23,3 ± 1,7	29	22,7 ± 1,3	RIA	ng/ml	mean-sem	plasma	case-control	prospective	30,6 ± 0,8	30,6 ± 0,9	31,4 ± 1	28,3 ± 0,6	N/
Buhling et al.	2005	Germany	Europe	34	38,9 ± 28,5	61	23,7 ± 14,8	ELISA	ng/ml	mean-sd	plasma	case-control	retrospective	29,3 ± 5,7	29 ± 5,5	32,4 ± 7,2	27,9 ± 3,6	N/
Atègbo et al	2006	France	Europe	59	0,59 ± 0,03	60	0,39 ± 0,03	ELISA	ng/ml	raw (mean-sd)	Serum	case-control	prospective	N/A	N/A	N/A	N/A	6,
McLachlan et al	2006	Australia	Oceania	19	23 ± 10,03	19	32,2 ± 17	RIA	ng/ml	mean-sem	plasma	case-control	prospective	33 ± 1	33 ± 1	31,5 ± 1,3	31,6 ± 1,3	5,
Maghbooli et al.	2007	Iran	Asia	52	29,05 ± 8,57	527	20,35 ± 15,15	ELISA	ng/ml	mean-sd	Serum	case-control	retrospective	30,23 ± 5,7	25,14 ± 4,44	28,5 ± 4,73	24,44 ± 4,8	5,
Palik et al.	2007	Hungary	Europe	30	40,32 ± 24,46	15	34,91 ± 19,4	ELISA	ng/ml	mean-sd	Serum	case-control	retrospective	28,12 ± 2,71	27,2 ± 2,9	32,68 ± 6,02	28,8 ± 5,2	4,
Georgiou et al.	2008	Australia	Oceania	14	26,1 ± 16,5	14	21,2 ± 14,5	ELISA	ng/ml	mean-sd	plasma	case-control	prospective	33,8 ± 5	32,6 ± 3,4	28,2 ± 8,4	24,7 ± 5,1	5,
Şengül et al.	2009	Turkey	Asia	20	9,1 ± 5,3	20	6,3 ± 4,6	ELISA	ng/ml	mean-sd	Serum	case-control	retrospective	29,6 ± 5,8	27,5 ± 4,2	29 ± 3,2	26,2 ± 3,6	N/
Retnakaran et al.	2010	Canada	North America	137	34,82 ± 13,49	259	35,11 ± 19,46	ELISA	ng/ml	median-iqr	Serum	case-control	prospective	34,5 ± 4,3	33,9 ± 4,3	N/A	N/A	N/
Horosz et al.	2011	Poland	Europe	86	24,14 ± 12,77	48	21,34 ± 11,67	ELISA	ng/ml	mean-sd	Serum	case-control	prospective	31,8 ± 4,3	30,7 ± 3,5	28,39 ± 5,32	25,4 ± 2,72	4,
Mokhtari et al.	2011	Iran	Asia	26	9,89 ± 7,764	22	13,8 ± 10,32	ELISA	ng/ml	mean-sd	N/A	case-control	prospective	32,69 ± 6,85	28,18 ± 8,93	28,51 ± 3,66	27,24 ± 4,06	N/
Saucedo et al	2011	Mexico	Central America	60	22,7 ± 4,9	60	23,3 ± 11,7	RIA	ng/ml	mean-sd	N/A	case-control	prospective	31,9 ± 5,6	24,8 ± 6,4	30,2 ± 4,9	28,4 ± 7,3	5,
López-Tinoco et al.	2012	Spain	Europe	56	27,37 ± 16,21	48	18,72 ± 8,86	ELISA	1pg/ml=0,001 ng/ml	mean-sd	plasma	case-control	prospective	31,57 ± 4,1	30,52 ± 4,5	27,1 ± 4,6	23,31 ± 4,2	5,
Maple-Brown et al.	2012	Canada	North America	198	37,77 ± 18,15	477	35,21 ± 18,96	ELISA	ng/ml	median-iqr	Serum	case-control	prospective	34,6 ± 4,5	34 ± 4,2	N/A	N/A	N/
Skvarca et al.	2012	Slovenia	Europe	30	24,84 ± 18,62	25	22,32 ± 13,38	ELISA	mcg/L= ng/ml	mean-sd	Serum	case-control	retrospective	30,33 ± 4,86	31,2 ± 3,34	27,39 ± 3,8	25,33 ± 3,34	5,
Boyadzhieva et al.	2013	Bulgaria	Europe	127	54,27 ± 10,35	109	20,4 ± 7,7	ELISA	ng/ml	mean-sd	Serum	case-control	prospective	32,2 ± 5,2	30,6 ± 4,4	28,5 ± 6,6	28 ± 5,4	5,
McManus et al.	2014	Canada	North America	36	40,1 ± 26,4	37	41,2 ± 33,7	ELISA	ng/ml	mean-sd	Plasma	case-control	prospective	31,6 ± 5	30,2 ± 4,1	32,91 ± 5,06	31,37 ± 8,11	5,
Noureldeen et al.(cohort1)	2014	Saudi Arabia	Asia	24	3,12 ± 0,3	33	3,72 ± 0,315	ELISA	ng/ml	mean-sd	Serum	case-control	retospective	36,38 ± 1,007	27,65 ± 1,041	31,1 ± 1,066	27,65 ± 1,284	5,
Noureldeen et al.(cohort2)	2014	Saudi Arabia	Asia	47	4,25 ± 0,43	38	7,25 ± 0,819	ELISA	ng/ml	mean-sd	Serum	case-control	retospective	33,51 ± 0,872	31,16 ± 1,022	30,08 ± 0,736	27,13 ± 0,817	5,
Fruscalzo et al.	2015	Germany	Europe	8	0,15 ± 0,153	10	0,17 ± 0,126	ELISA	1pg/ml=0,001 ng/ml	median-iqr	Serum	case-control	prospective	36,9 ± 1,5	38 ± 1,3	N/A	N/A	5,
Saini et al.	2015	India	Asia	30	42,96 ± 19,47	60	16,53 ± 7,58	ELISA	ng/ml	mean-sd	N/A	case-control	prospective	N/A	N/A	N/A	N/A	9,
Martino et al.	2016	Spain	Europe	25	27,92 ± 19,3	110	21,74 ± 19,54	ELISA	mcg/L= ng/ml	mean-sd (subgroups)	Serum	case-control	prospective	33,8 ± 4,18	30,25 ± 5,37	30,52 ± 6,24	29,6 ± 4,32	6,
Zhang et al.	2016	China	Asia	40	10,57 ± 2,46	240	4,71 ± 1,29	ELISA	mcg/L= ng/ml	mean-sd	Serum	case-control	prospective	32,24 ± 3,81	28,21 ± 4,12	23,6 ± 3,5	20,83 ± 3,01	5,
Fatima et al.	2017	Pakistan	Asia	208	20,38 ± 12,7	300	3,41 ± 2,17	ELISA	ng/ml	mean-sd	Serum	case-control	prospective	27,3 ± 5,56	25,78 ± 4,73	24,83 ± 5,13	22,38 ± 3,93	5,
Zhang et al.	2017	China	Asia	50	9,24 ± 1,94	50	5,28 ± 1,29	ELISA	mcg/L= ng/ml	mean-sd	Serum	case-control	prospective	31,78 ± 4,81	30,16 ± 4,46	27,5 ± 3,91	26,98 ± 3,34	4,
Al-Daghri et al.	2018	Saudi Arabia	Asia	63	1,58 ± 2,82	54	1,36706 ± 2,38167	ELISA	1pg/ml=0,001 ng/ml	median-iqr	Serum	case-control	retospective	26,1 ± 3,6	26,4 ± 3	29,7 ± 6,6	26,7 ± 5,7	5,
Mosavat et al.	2018	Malaysia	Asia	53	19,8 ± 1,7	43	26,6 ± 2,7	ELISA	ng/ml	mean-sd	Serum	case-control	prospective	33,15 ± 0,59	32,05 ± 0,75	29,26 ± 0,7	29,09 ± 0,9	N/
Bawah et al.	2019	Ghana	Africa	70	35,04 ± 8,7	70	21,94 ± 9,19	ELISA	ng/ml	mean-sd	Serum	case-control	prospective	30,87 ± 5,74	28,76 ± 5,09	27,53 ± 3,93	25,58 ± 3,84	N/
Ebert et al.	2020	Germany	Europe	74	26,78 ± 16,33	74	23,39 ± 9	ELISA	mcg/L= ng/ml	median-iqr	Serum	case-control	retrospective	30,61 ± 5,52	28,35 ± 3,78	24,5 ± 4,96	23,61 ± 5,08	4,
Schuitemaker et al.	2020	Netherlands	Europe	50	1,44 ± 0,28	100	1,32 ± 0,24	ELISA	ng/ml	mean-sd	Serum	case-control	prospective	34,68 ± 4,2	34,38 ± 5,12	26,42 ± 5,73	26,02 ± 5,49	N/
Tenenbaum-Gavish et al.	2020	Israel	Asia	20	3,87 ± 1,57	185	1,94 ± 0,67	ELISA	1pg/ml=0,001 ng/ml	median-iqr	Serum	case-control	prospective	33,4 ± 4,31	31 ± 0,97	30 ± 4,79	23,3 ± 0,82	N/
Wan-Qing Xiao et al.	2020	China	Asia	198	19,48 ± 9,91	192	15,91 ± 9,81	ELISA	ng/ml	median-iqr	plasma	case-control	prospective	30,5 ± 3,4	28,8 ± 3,2	N/A	N/A	N/
Al-Musharaf et al.	2021	Saudi Arabia	Asia	99	2,6 ± 4,6	133	3,6 ± 5,1	ELISA	ng/ml	mean-sd	Serum	case-control	prospective	29,6 ± 5,4	30,2 ± 4,5	29,2 ± 5,5	29,5 ± 5,9	N/
Florian et al.	2021	Romania	Europe	21	35,47 ± 18,37	47	19,6 ± 17,21	ELISA	ng/ml	median-iqr	Serum	case-control	prospective	32,54 ± 4,37	30 ± 4,59	24,7 ± 5,65	21,64 ± 3,29	N/

GDM, gestational diabetes mellitus; BMI, body mass index (kg/m2); FPG, fasting plasma glucose (mmol/l); N/A, not available; **gestational age at delivery (weeks); Caesarean (%); newborn weight (grams).**

4. Subgroup meta-analyses

4.1 Geographical area

Figure S1. Forest plot graphically representing the subgroup meta-analysis evaluating the changes in circulating leptin levels between GDM patients and healthy control women, stratified by geographical area.

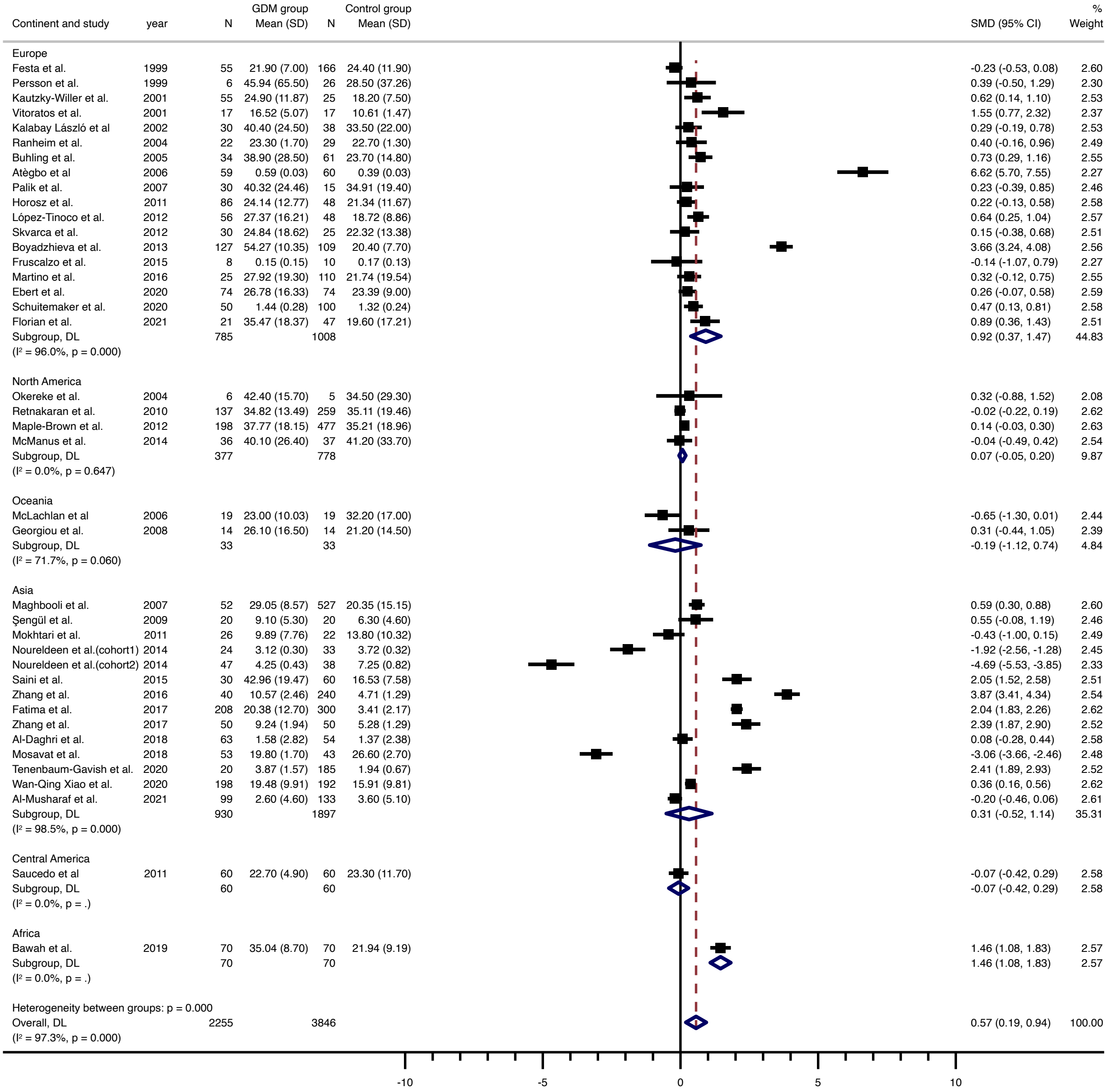

Random-effects model, inverse-variance weighting based on the DerSimonian and Laird method. Standardized mean difference (SMD) was chosen as effect size measure. A SMD>0 suggests that leptin levels are higher in GDM. Diamonds indicate the overall pooled SMDs with their corresponding 95% confidence intervals (CI).

4.2 Trimester

Figure S2. Forest plot graphically representing the subgroup meta-analysis evaluating the changes in circulating leptin levels between GDM patients and healthy control women, stratified by trimester (first vs second vs third).

Trimester and study	year	N	GDM group Mean (SD)	N	Control group Mean (SD)	SMD (95% CI)	% Weight
1.first							
Georgiou et al.	2008	14	26.10 (16.50)	14	21.20 (14.50)	0.31 (-0.44, 1.05)	2.39
Okereke et al.	2004	6	42.40 (15.70)	5	34.50 (29.30)	0.32 (-0.88, 1.52)	2.08
Al-Musharaf et al.	2021	99	2.60 (4.60)	133	3.60 (5.10)	-0.20 (-0.46, 0.06)	2.61
Schuitemaker et al.	2020	50	1.44 (0.28)	100	1.32 (0.24)	0.47 (0.13, 0.81)	2.58
Bawah et al.	2019	70	35.04 (8.70)	70	21.94 (9.19)	1.46 (1.08, 1.83)	2.57
Tenenbaum-Gavish et al.	2020	20	3.87 (1.57)	185	1.94 (0.67)	2.41 (1.89, 2.93)	2.52
Subgroup, DL		259		507		0.81 (-0.02, 1.64)	14.75
(I^2 = 95.2%, p = 0.000)							
2.second							
Fruscalzo et al.	2015	8	0.15 (0.15)	10	0.17 (0.13)	-0.14 (-1.07, 0.79)	2.27
Fatima et al.	2017	208	20.38 (12.70)	300	3.41 (2.17)	2.04 (1.83, 2.26)	2.62
Wan-Qing Xiao et al.	2020	198	19.48 (9.91)	192	15.91 (9.81)	0.36 (0.16, 0.56)	2.62
McManus et al.	2014	36	40.10 (26.40)	37	41.20 (33.70)	-0.04 (-0.49, 0.42)	2.54
Mokhtari et al.	2011	26	9.89 (7.76)	22	13.80 (10.32)	-0.43 (-1.00, 0.15)	2.49
Boyadzhieva et al.	2013	127	54.27 (10.35)	109	20.40 (7.70)	3.66 (3.24, 4.08)	2.56
Festa et al.	1999	55	21.90 (7.00)	166	24.40 (11.90)	-0.23 (-0.53, 0.08)	2.60
Mosavat et al.	2018	53	19.80 (1.70)	43	26.60 (2.70)	-3.06 (-3.66, -2.46)	2.48
Zhang et al.	2016	40	10.57 (2.46)	240	4.71 (1.29)	3.87 (3.41, 4.34)	2.54
Noureldeen et al.(cohort1)	2014	24	3.12 (0.30)	33	3.72 (0.32)	-1.92 (-2.56, -1.28)	2.45
Maghbooli et al.	2007	52	29.05 (8.57)	527	20.35 (15.15)	0.59 (0.30, 0.88)	2.60
Subgroup, DL		827		1679		0.44 (-0.52, 1.41)	27.76
(I^2 = 98.7%, p = 0.000)							
3.third							
Atègbo et al	2006	59	0.59 (0.03)	60	0.39 (0.03)	6.62 (5.70, 7.55)	2.27
Noureldeen et al.(cohort2)	2014	47	4.25 (0.43)	38	7.25 (0.82)	-4.69 (-5.53, -3.85)	2.33
Şengül et al.	2009	20	9.10 (5.30)	20	6.30 (4.60)	0.55 (-0.08, 1.19)	2.46
Buhling et al.	2005	34	38.90 (28.50)	61	23.70 (14.80)	0.73 (0.29, 1.16)	2.55
Zhang et al.	2017	50	9.24 (1.94)	50	5.28 (1.29)	2.39 (1.87, 2.90)	2.52
Al-Daghri et al.	2018	63	1.58 (2.82)	54	1.37 (2.38)	0.08 (-0.28, 0.44)	2.58
Persson et al.	1999	6	45.94 (65.50)	26	28.50 (37.26)	0.39 (-0.50, 1.29)	2.30
López-Tinoco et al.	2012	56	27.37 (16.21)	48	18.72 (8.86)	0.64 (0.25, 1.04)	2.57
Saucedo et al	2011	60	22.70 (4.90)	60	23.30 (11.70)	-0.07 (-0.42, 0.29)	2.58
Saini et al.	2015	30	42.96 (19.47)	60	16.53 (7.58)	2.05 (1.52, 2.58)	2.51
Retnakaran et al.	2010	137	34.82 (13.49)	259	35.11 (19.46)	-0.02 (-0.22, 0.19)	2.62
Horosz et al.	2011	86	24.14 (12.77)	48	21.34 (11.67)	0.22 (-0.13, 0.58)	2.58
Kautzky-Willer et al.	2001	55	24.90 (11.87)	25	18.20 (7.50)	0.62 (0.14, 1.10)	2.53
Palik et al.	2007	30	40.32 (24.46)	15	34.91 (19.40)	0.23 (-0.39, 0.85)	2.46
McLachlan et al	2006	19	23.00 (10.03)	19	32.20 (17.00)	-0.65 (-1.30, 0.01)	2.44
Vitoratos et al.	2001	17	16.52 (5.07)	17	10.61 (1.47)	1.55 (0.77, 2.32)	2.37
Ranheim et al.	2004	22	23.30 (1.70)	29	22.70 (1.30)	0.40 (-0.16, 0.96)	2.49
Maple-Brown et al.	2012	198	37.77 (18.15)	477	35.21 (18.96)	0.14 (-0.03, 0.30)	2.63
Kalabay László et al	2002	30	40.40 (24.50)	38	33.50 (22.00)	0.29 (-0.19, 0.78)	2.53
Skvarca et al.	2012	30	24.84 (18.62)	25	22.32 (13.38)	0.15 (-0.38, 0.68)	2.51
Ebert et al.	2020	74	26.78 (16.33)	74	23.39 (9.00)	0.26 (-0.07, 0.58)	2.59
Subgroup, DL		1123		1503		0.55 (0.12, 0.98)	52.43
(I^2 = 95.7%, p = 0.000)							
NR							
Florian et al.	2021	21	35.47 (18.37)	47	19.60 (17.21)	0.89 (0.36, 1.43)	2.51
Martino et al.	2016	25	27.92 (19.30)	110	21.74 (19.54)	0.32 (-0.12, 0.75)	2.55
Subgroup, DL		46		157		0.58 (0.02, 1.15)	5.06
(I^2 = 62.7%, p = 0.102)							
Heterogeneity between groups: p = 0.942							
Overall, DL		2255		3846		0.57 (0.19, 0.94)	100.00
(I^2 = 97.3%, p = 0.000)							

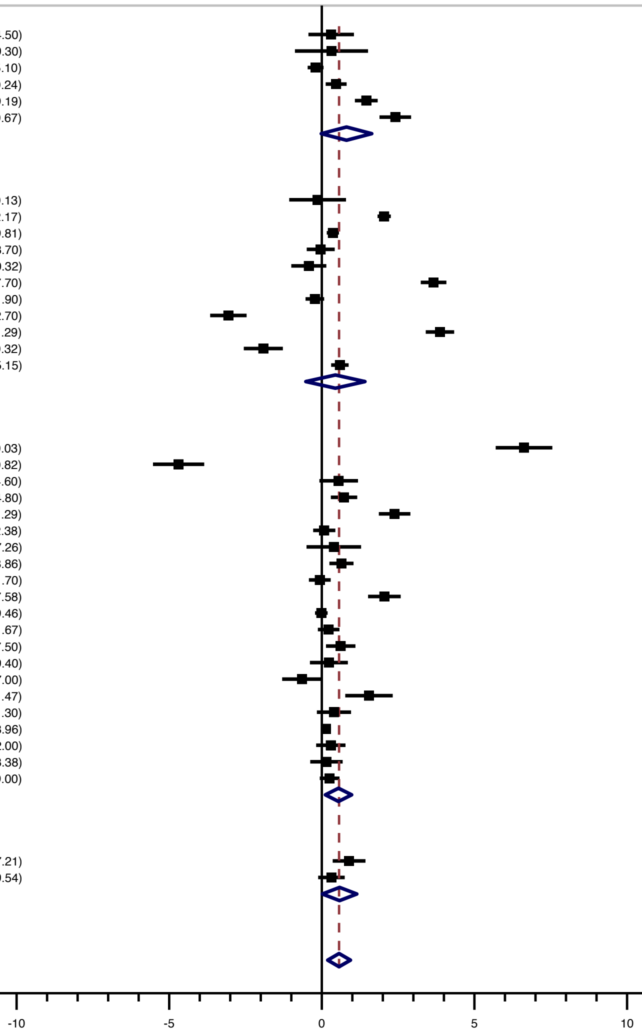

Random-effects model, inverse-variance weighting based on the DerSimonian and Laird method. Standardized mean difference (SMD) was chosen as effect size measure. A SMD>0 suggests that leptin levels are higher in GDM. Diamonds indicate the overall pooled SMDs with their corresponding 95% confidence intervals (CI).

4.3 Trimester

Figure S3. Forest plot graphically representing the subgroup meta-analysis evaluating the changes in circulating leptin levels between GDM patients and healthy control women, stratified by trimester (first vs other).

firstTrimester and study	year	GDM group N	GDM group Mean (SD)	Control group N	Control group Mean (SD)	SMD (95% CI)	% Weight
1.first							
Georgiou et al.	2008	14	26.10 (16.50)	14	21.20 (14.50)	0.31 (-0.44, 1.05)	2.39
Okereke et al.	2004	6	42.40 (15.70)	5	34.50 (29.30)	0.32 (-0.88, 1.52)	2.08
Al-Musharaf et al.	2021	99	2.60 (4.60)	133	3.60 (5.10)	-0.20 (-0.46, 0.06)	2.61
Schuitemaker et al.	2020	50	1.44 (0.28)	100	1.32 (0.24)	0.47 (0.13, 0.81)	2.58
Bawah et al.	2019	70	35.04 (8.70)	70	21.94 (9.19)	1.46 (1.08, 1.83)	2.57
Tenenbaum-Gavish et al.	2020	20	3.87 (1.57)	185	1.94 (0.67)	2.41 (1.89, 2.93)	2.52
Subgroup, DL		259		507		0.81 (-0.02, 1.64)	14.75
(I² = 95.2%, p = 0.000)							
other							
Fruscalzo et al.	2015	8	0.15 (0.15)	10	0.17 (0.13)	-0.14 (-1.07, 0.79)	2.27
Fatima et al.	2017	208	20.38 (12.70)	300	3.41 (2.17)	2.04 (1.83, 2.26)	2.62
Wan-Qing Xiao et al.	2020	198	19.48 (9.91)	192	15.91 (9.81)	0.36 (0.16, 0.56)	2.62
McManus et al.	2014	36	40.10 (26.40)	37	41.20 (33.70)	-0.04 (-0.49, 0.42)	2.54
Mokhtari et al.	2011	26	9.89 (7.76)	22	13.80 (10.32)	-0.43 (-1.00, 0.15)	2.49
Boyadzhieva et al.	2013	127	54.27 (10.35)	109	20.40 (7.70)	3.66 (3.24, 4.08)	2.56
Festa et al.	1999	55	21.90 (7.00)	166	24.40 (11.90)	-0.23 (-0.53, 0.08)	2.60
Mosavat et al.	2018	53	19.80 (1.70)	43	26.60 (2.70)	-3.06 (-3.66, -2.46)	2.48
Zhang et al.	2016	40	10.57 (2.46)	240	4.71 (1.29)	3.87 (3.41, 4.34)	2.54
Noureldeen et al.(cohort1)	2014	24	3.12 (0.30)	33	3.72 (0.32)	-1.92 (-2.56, -1.28)	2.45
Maghbooli et al.	2007	52	29.05 (8.57)	527	20.35 (15.15)	0.59 (0.30, 0.88)	2.60
Atègbo et al	2006	59	0.59 (0.03)	60	0.39 (0.03)	6.62 (5.70, 7.55)	2.27
Noureldeen et al.(cohort2)	2014	47	4.25 (0.43)	38	7.25 (0.82)	-4.69 (-5.53, -3.85)	2.33
Şengül et al.	2009	20	9.10 (5.30)	20	6.30 (4.60)	0.55 (-0.08, 1.19)	2.46
Buhling et al.	2005	34	38.90 (28.50)	61	23.70 (14.80)	0.73 (0.29, 1.16)	2.55
Zhang et al.	2017	50	9.24 (1.94)	50	5.28 (1.29)	2.39 (1.87, 2.90)	2.52
Al-Daghri et al.	2018	63	1.58 (2.82)	54	1.37 (2.38)	0.08 (-0.28, 0.44)	2.58
Persson et al.	1999	6	45.94 (65.50)	26	28.50 (37.26)	0.39 (-0.50, 1.29)	2.30
López-Tinoco et al.	2012	56	27.37 (16.21)	48	18.72 (8.86)	0.64 (0.25, 1.04)	2.57
Saucedo et al	2011	60	22.70 (4.90)	60	23.30 (11.70)	-0.07 (-0.42, 0.29)	2.58
Saini et al.	2015	30	42.96 (19.47)	60	16.53 (7.58)	2.05 (1.52, 2.58)	2.51
Retnakaran et al.	2010	137	34.82 (13.49)	259	35.11 (19.46)	-0.02 (-0.22, 0.19)	2.62
Horosz et al.	2011	86	24.14 (12.77)	48	21.34 (11.67)	0.22 (-0.13, 0.58)	2.58
Kautzky-Willer et al.	2001	55	24.90 (11.87)	25	18.20 (7.50)	0.62 (0.14, 1.10)	2.53
Palik et al.	2007	30	40.32 (24.46)	15	34.91 (19.40)	0.23 (-0.39, 0.85)	2.46
McLachlan et al	2006	19	23.00 (10.03)	19	32.20 (17.00)	-0.65 (-1.30, 0.01)	2.44
Vitoratos et al.	2001	17	16.52 (5.07)	17	10.61 (1.47)	1.55 (0.77, 2.32)	2.37
Ranheim et al.	2004	22	23.30 (1.70)	29	22.70 (1.30)	0.40 (-0.16, 0.96)	2.49
Maple-Brown et al.	2012	198	37.77 (18.15)	477	35.21 (18.96)	0.14 (-0.03, 0.30)	2.63
Kalabay László et al	2002	30	40.40 (24.50)	38	33.50 (22.00)	0.29 (-0.19, 0.78)	2.53
Skvarca et al.	2012	30	24.84 (18.62)	25	22.32 (13.38)	0.15 (-0.38, 0.68)	2.51
Ebert et al.	2020	74	26.78 (16.33)	74	23.39 (9.00)	0.26 (-0.07, 0.58)	2.59
Subgroup, DL		1950		3182		0.52 (0.08, 0.96)	80.19
(I² = 97.7%, p = 0.000)							
NR							
Florian et al.	2021	21	35.47 (18.37)	47	19.60 (17.21)	0.89 (0.36, 1.43)	2.51
Martino et al.	2016	25	27.92 (19.30)	110	21.74 (19.54)	0.32 (-0.12, 0.75)	2.55
Subgroup, DL		46		157		0.58 (0.02, 1.15)	5.06
(I² = 62.7%, p = 0.102)							
Heterogeneity between groups: p = 0.833							
Overall, DL		2255		3846		0.57 (0.19, 0.94)	100.00
(I² = 97.3%, p = 0.000)							

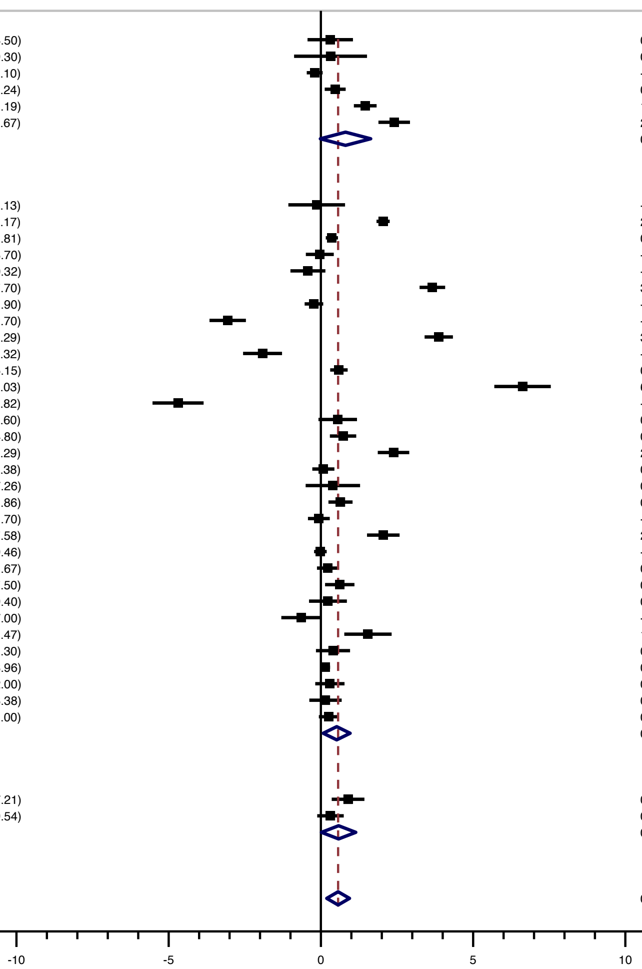

Random-effects model, inverse-variance weighting based on the DerSimonian and Laird method. Standardized mean difference (SMD) was chosen as effect size measure. A SMD>0 suggests that leptin levels are higher in GDM. Diamonds indicate the overall pooled SMDs with their corresponding 95% confidence intervals (CI).

4.4 Trimester

Figure S4. Forest plot graphically representing the subgroup meta-analysis evaluating the changes in circulating leptin levels between GDM patients and healthy control women, stratified by trimester (second vs other).

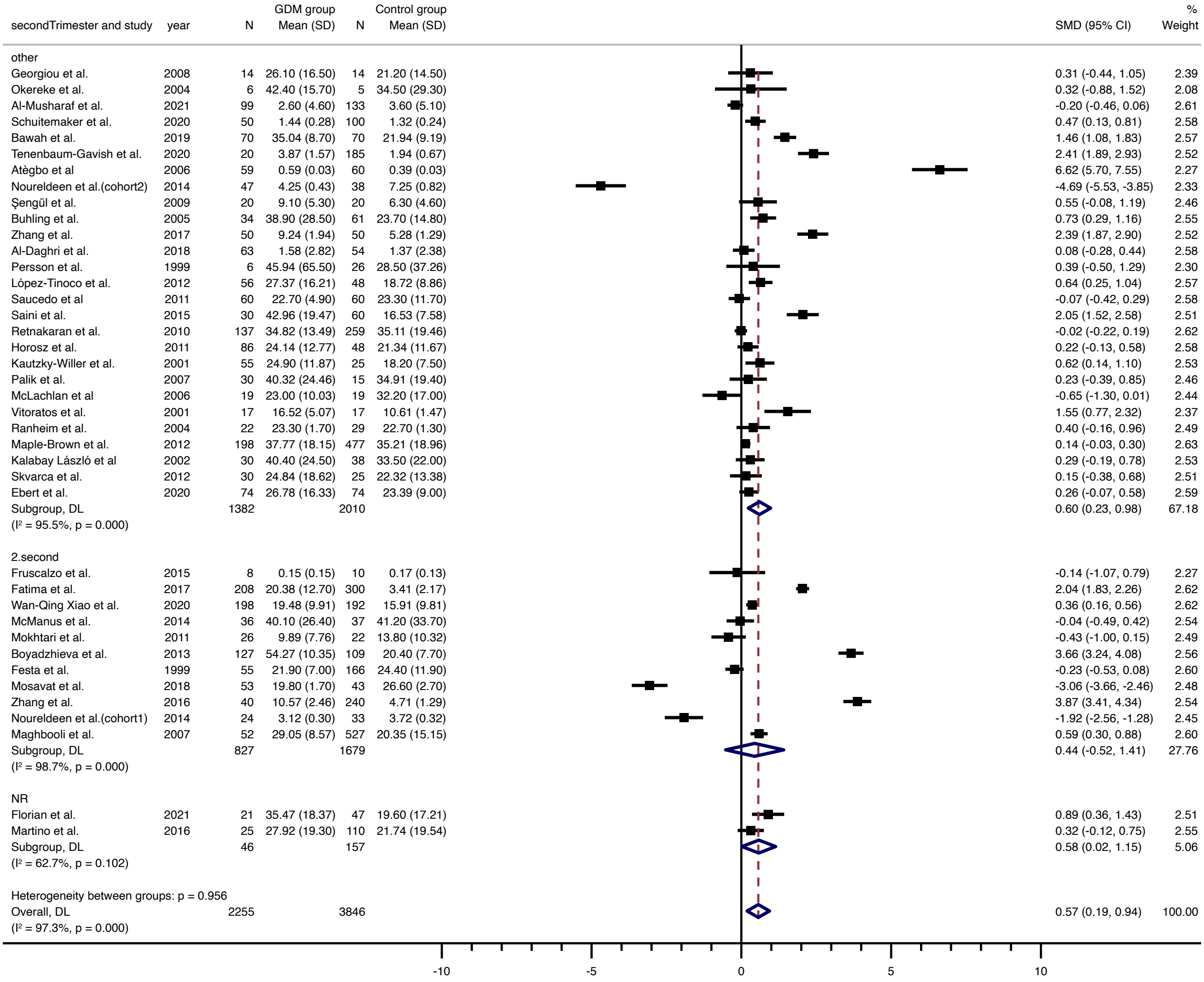

Random-effects model, inverse-variance weighting based on the DerSimonian and Laird method. Standardized mean difference (SMD) was chosen as effect size measure. A SMD>0 suggests that leptin levels are higher in GDM. Diamonds indicate the overall pooled SMDs with their corresponding 95% confidence intervals (CI).

4.5 Trimester

Figure S5. Forest plot graphically representing the subgroup meta-analysis evaluating the changes in circulating leptin levels between GDM patients and healthy control women, stratified by trimester (third vs other).

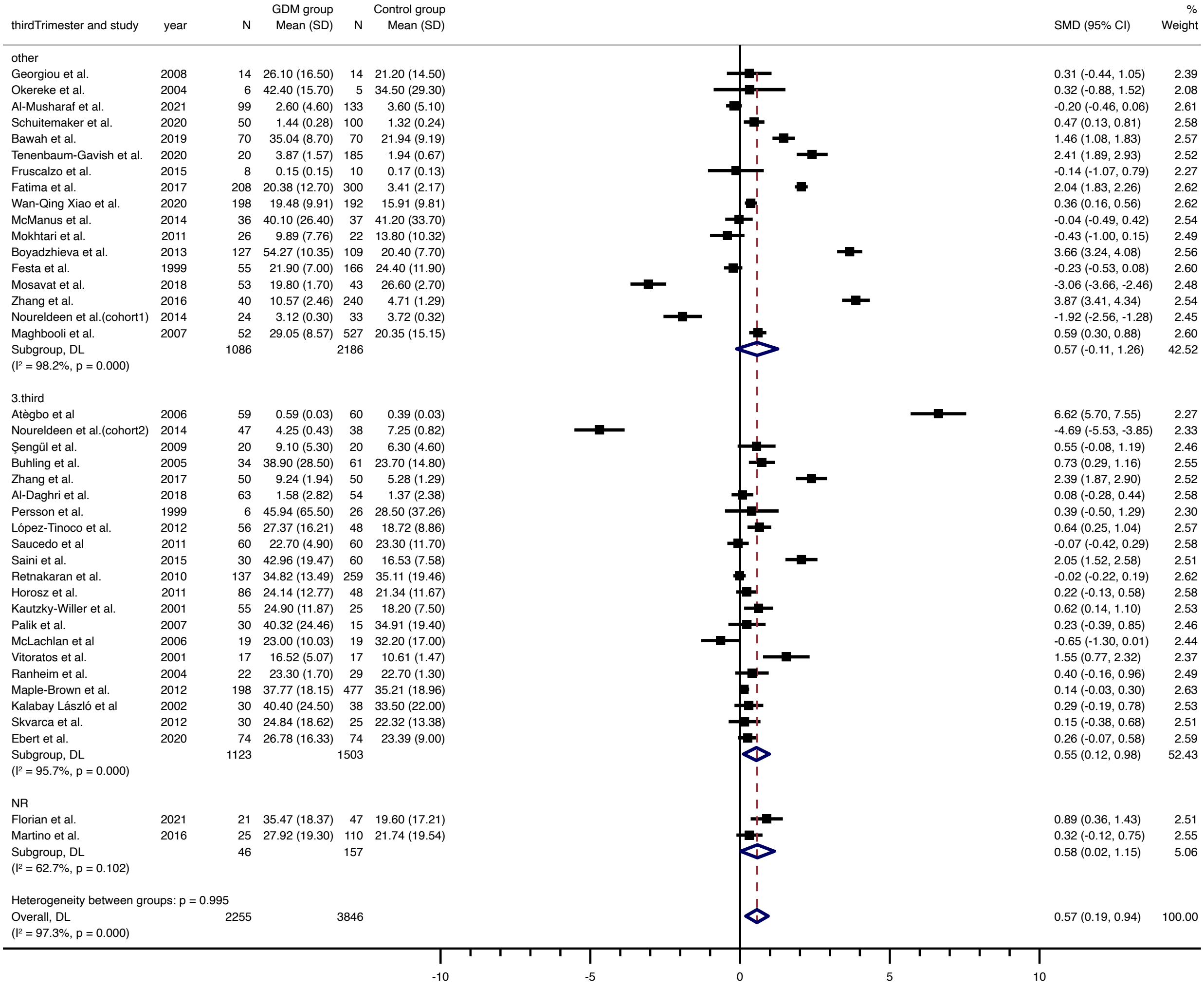

Random-effects model, inverse-variance weighting based on the DerSimonian and Laird method. Standardized mean difference (SMD) was chosen as effect size measure. A SMD>0 suggests that leptin levels are higher in GDM. Diamonds indicate the overall pooled SMDs with their corresponding 95% confidence intervals (CI).

4.6 Type of analysis

Figure S6. Forest plot graphically representing the subgroup meta-analysis evaluating the changes in circulating leptin levels between GDM patients and healthy control women, stratified by type of analysis (ELISA vs RIA).

Technique and study	year	GDM group N	GDM group Mean (SD)	Control group N	Control group Mean (SD)	SMD (95% CI)	% Weight
RIA							
Festa et al.	1999	55	21.90 (7.00)	166	24.40 (11.90)	-0.23 (-0.54, 0.08)	2.60
Persson et al.	1999	6	45.94 (65.50)	26	28.50 (37.26)	0.40 (-0.49, 1.30)	2.30
Kautzky-Willer et al.	2001	55	24.90 (11.87)	25	18.20 (7.50)	0.63 (0.14, 1.11)	2.53
Okereke et al.	2004	6	42.40 (15.70)	5	34.50 (29.30)	0.35 (-0.85, 1.54)	2.08
Ranheim et al.	2004	22	23.30 (1.70)	29	22.70 (1.30)	0.40 (-0.16, 0.96)	2.49
McLachlan et al	2006	19	23.00 (10.03)	19	32.20 (17.00)	-0.66 (-1.31, -0.01)	2.45
Saucedo et al	2011	60	22.70 (4.90)	60	23.30 (11.70)	-0.07 (-0.42, 0.29)	2.58
Subgroup, DL		223		330		0.07 (-0.26, 0.40)	17.03
(I^2 = 61.9%, p = 0.015)							
ELISA							
Vitoratos et al.	2001	17	16.52 (5.07)	17	10.61 (1.47)	1.58 (0.81, 2.36)	2.37
Kalabay László et al	2002	30	40.40 (24.50)	38	33.50 (22.00)	0.30 (-0.18, 0.78)	2.53
Buhling et al.	2005	34	38.90 (28.50)	61	23.70 (14.80)	0.73 (0.30, 1.17)	2.55
Atègbo et al	2006	59	0.59 (0.03)	60	0.39 (0.03)	6.67 (5.74, 7.59)	2.28
Maghbooli et al.	2007	52	29.05 (8.57)	527	20.35 (15.15)	0.59 (0.31, 0.88)	2.60
Palik et al.	2007	30	40.32 (24.46)	15	34.91 (19.40)	0.24 (-0.39, 0.86)	2.46
Georgiou et al.	2008	14	26.10 (16.50)	14	21.20 (14.50)	0.32 (-0.43, 1.06)	2.39
Şengül et al.	2009	20	9.10 (5.30)	20	6.30 (4.60)	0.56 (-0.07, 1.20)	2.46
Retnakaran et al.	2010	137	34.82 (13.49)	259	35.11 (19.46)	-0.02 (-0.22, 0.19)	2.62
Horosz et al.	2011	86	24.14 (12.77)	48	21.34 (11.67)	0.23 (-0.13, 0.58)	2.58
Mokhtari et al.	2011	26	9.89 (7.76)	22	13.80 (10.32)	-0.43 (-1.01, 0.14)	2.49
López-Tinoco et al.	2012	56	27.37 (16.21)	48	18.72 (8.86)	0.65 (0.25, 1.04)	2.57
Maple-Brown et al.	2012	198	37.77 (18.15)	477	35.21 (18.96)	0.14 (-0.03, 0.30)	2.63
Skvarca et al.	2012	30	24.84 (18.62)	25	22.32 (13.38)	0.15 (-0.38, 0.68)	2.51
Boyadzhieva et al.	2013	127	54.27 (10.35)	109	20.40 (7.70)	3.67 (3.25, 4.09)	2.56
McManus et al.	2014	36	40.10 (26.40)	37	41.20 (33.70)	-0.04 (-0.50, 0.42)	2.54
Noureldeen et al.(cohort1)	2014	24	3.12 (0.30)	33	3.72 (0.32)	-1.94 (-2.58, -1.30)	2.45
Noureldeen et al.(cohort2)	2014	47	4.25 (0.43)	38	7.25 (0.82)	-4.73 (-5.57, -3.90)	2.34
Fruscalzo et al.	2015	8	0.15 (0.15)	10	0.17 (0.13)	-0.14 (-1.08, 0.79)	2.27
Saini et al.	2015	30	42.96 (19.47)	60	16.53 (7.58)	2.07 (1.53, 2.60)	2.51
Martino et al.	2016	25	27.92 (19.30)	110	21.74 (19.54)	0.32 (-0.12, 0.75)	2.55
Zhang et al.	2016	40	10.57 (2.46)	240	4.71 (1.29)	3.88 (3.42, 4.35)	2.54
Fatima et al.	2017	208	20.38 (12.70)	300	3.41 (2.17)	2.05 (1.83, 2.26)	2.62
Zhang et al.	2017	50	9.24 (1.94)	50	5.28 (1.29)	2.40 (1.89, 2.92)	2.52
Al-Daghri et al.	2018	63	1.58 (2.82)	54	1.37 (2.38)	0.08 (-0.28, 0.44)	2.58
Mosavat et al.	2018	53	19.80 (1.70)	43	26.60 (2.70)	-3.09 (-3.68, -2.49)	2.48
Bawah et al.	2019	70	35.04 (8.70)	70	21.94 (9.19)	1.46 (1.09, 1.84)	2.57
Ebert et al.	2020	74	26.78 (16.33)	74	23.39 (9.00)	0.26 (-0.07, 0.58)	2.59
Schuitemaker et al.	2020	50	1.44 (0.28)	100	1.32 (0.24)	0.47 (0.13, 0.82)	2.58
Tenenbaum-Gavish et al.	2020	20	3.87 (1.57)	185	1.94 (0.67)	2.42 (1.90, 2.93)	2.51
Wan-Qing Xiao et al.	2020	198	19.48 (9.91)	192	15.91 (9.81)	0.36 (0.16, 0.56)	2.62
Al-Musharaf et al.	2021	99	2.60 (4.60)	133	3.60 (5.10)	-0.20 (-0.47, 0.06)	2.61
Florian et al.	2021	21	35.47 (18.37)	47	19.60 (17.21)	0.90 (0.37, 1.44)	2.51
Subgroup, DL		2032		3516		0.66 (0.23, 1.10)	82.97
(I^2 = 97.7%, p = 0.000)							
Heterogeneity between groups: p = 0.033							
Overall, DL		2255		3846		0.57 (0.19, 0.95)	100.00
(I^2 = 97.3%, p = 0.000)							

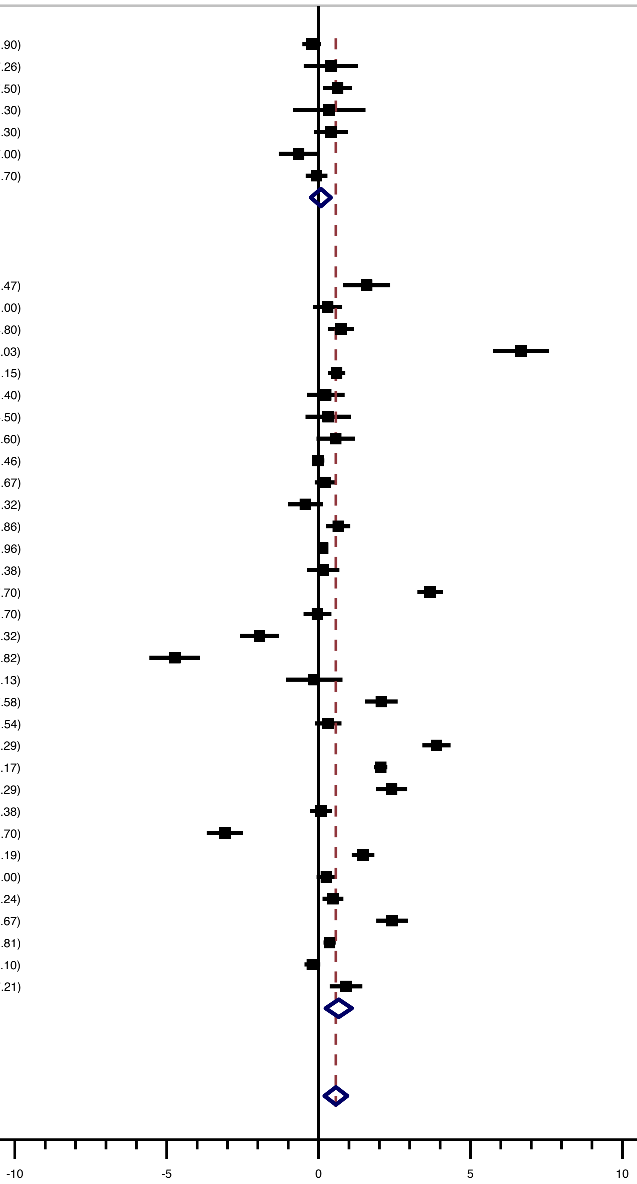

Random-effects model, inverse-variance weighting based on the DerSimonian and Laird method. Standardized mean difference (SMD) was chosen as effect size measure. A SMD>0 suggests that leptin levels are higher in GDM. Diamonds indicate the overall pooled SMDs with their corresponding 95% confidence intervals (CI).

4.7 Source of sample

Figure S7. Forest plot graphically representing the subgroup meta-analysis evaluating the changes in circulating leptin levels between GDM patients and healthy control women, stratified by type of analysis (plasma vs serum).

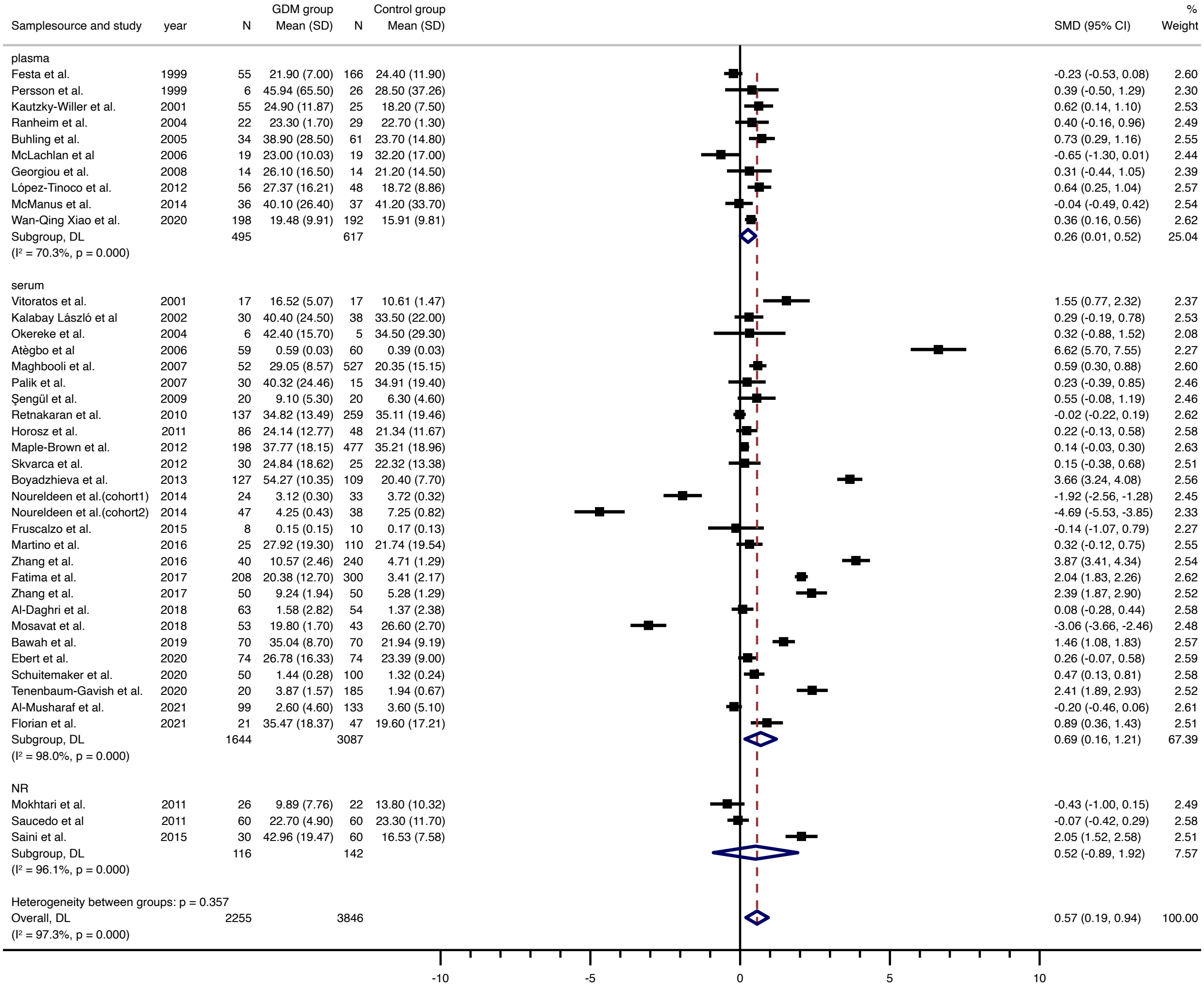

Random-effects model, inverse-variance weighting based on the DerSimonian and Laird method. Standardized mean difference (SMD) was chosen as effect size measure. A SMD>0 suggests that leptin levels are higher in GDM. Diamonds indicate the overall pooled SMDs with their corresponding 95% confidence intervals (CI).

4.8 Study design

Figure S8. Forest plot graphically representing the subgroup meta-analysis evaluating the changes in circulating leptin levels between GDM patients and healthy control women, stratified by study design (prospective vs. retrospective).

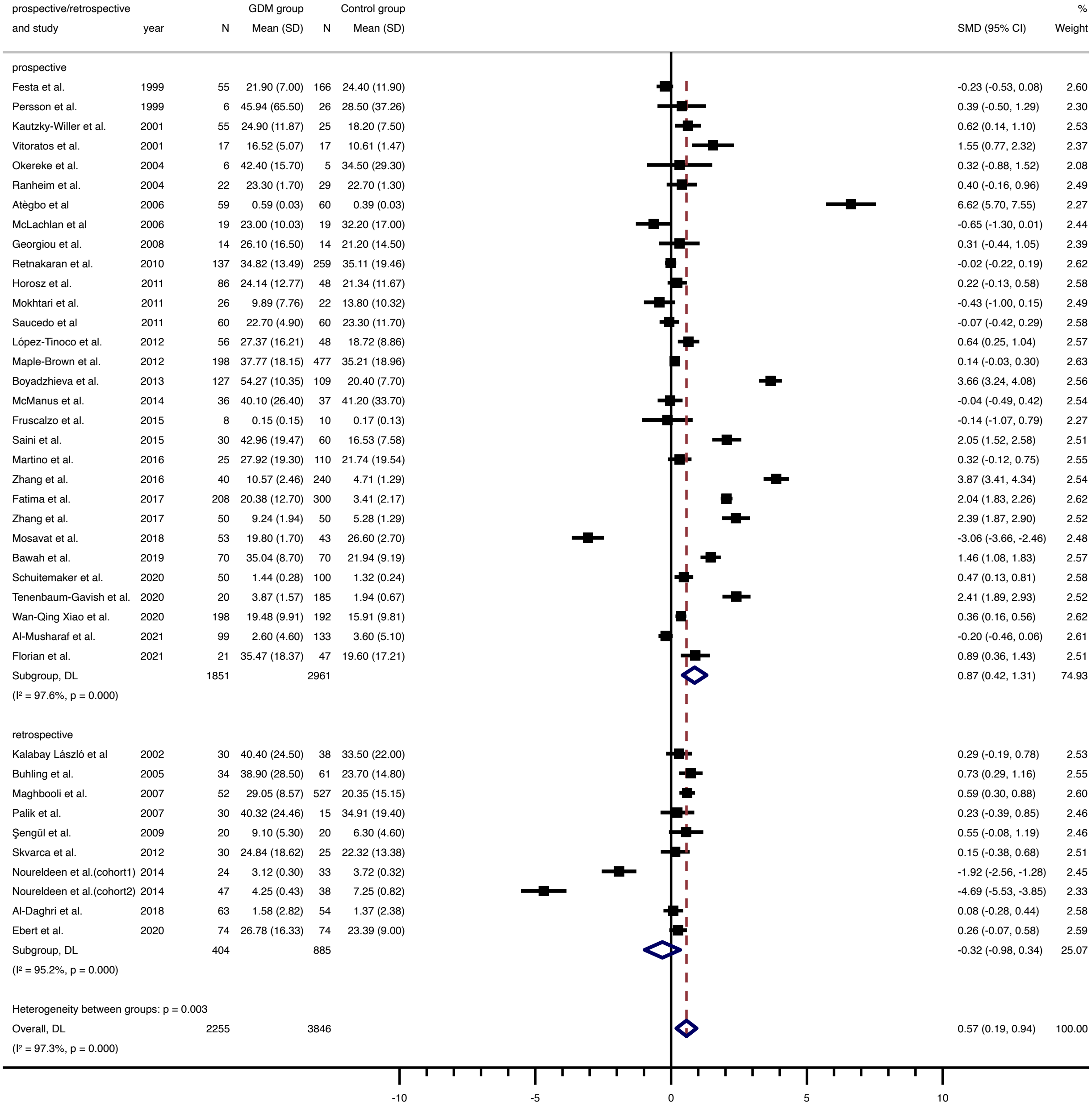

Random-effects model, inverse-variance weighting based on the DerSimonian and Laird method. Standardized mean difference (SMD) was chosen as effect size measure. A SMD>0 suggests that leptin levels are higher in GDM. Diamonds indicate the overall pooled SMDs with their corresponding 95% confidence intervals (CI).

4.9 Loss of patients assessment

Figure S9. Forest plot graphically representing the subgroup meta-analysis evaluating the changes in circulating leptin levels between GDM patients and healthy control women, stratified by the assessment of the loss of patients.

assess loss of patients and study	year	GDM group N	GDM group Mean (SD)	Control group N	Control group Mean (SD)	SMD (95% CI)	% Weight
no							
Festa et al.	1999	55	21.90 (7.00)	166	24.40 (11.90)	-0.23 (-0.53, 0.08)	2.60
Persson et al.	1999	6	45.94 (65.50)	26	28.50 (37.26)	0.39 (-0.50, 1.29)	2.30
Kautzky-Willer et al.	2001	55	24.90 (11.87)	25	18.20 (7.50)	0.62 (0.14, 1.10)	2.53
Kalabay László et al	2002	30	40.40 (24.50)	38	33.50 (22.00)	0.29 (-0.19, 0.78)	2.53
Okereke et al.	2004	6	42.40 (15.70)	5	34.50 (29.30)	0.32 (-0.88, 1.52)	2.08
Ranheim et al.	2004	22	23.30 (1.70)	29	22.70 (1.30)	0.40 (-0.16, 0.96)	2.49
Buhling et al.	2005	34	38.90 (28.50)	61	23.70 (14.80)	0.73 (0.29, 1.16)	2.55
Atègbo et al	2006	59	0.59 (0.03)	60	0.39 (0.03)	6.62 (5.70, 7.55)	2.27
McLachlan et al	2006	19	23.00 (10.03)	19	32.20 (17.00)	-0.65 (-1.30, 0.01)	2.44
Maghbooli et al.	2007	52	29.05 (8.57)	527	20.35 (15.15)	0.59 (0.30, 0.88)	2.60
Palik et al.	2007	30	40.32 (24.46)	15	34.91 (19.40)	0.23 (-0.39, 0.85)	2.46
Şengül et al.	2009	20	9.10 (5.30)	20	6.30 (4.60)	0.55 (-0.08, 1.19)	2.46
Retnakaran et al.	2010	137	34.82 (13.49)	259	35.11 (19.46)	-0.02 (-0.22, 0.19)	2.62
Horosz et al.	2011	86	24.14 (12.77)	48	21.34 (11.67)	0.22 (-0.13, 0.58)	2.58
Mokhtari et al.	2011	26	9.89 (7.76)	22	13.80 (10.32)	-0.43 (-1.00, 0.15)	2.49
Saucedo et al	2011	60	22.70 (4.90)	60	23.30 (11.70)	-0.07 (-0.42, 0.29)	2.58
Maple-Brown et al.	2012	198	37.77 (18.15)	477	35.21 (18.96)	0.14 (-0.03, 0.30)	2.63
Skvarca et al.	2012	30	24.84 (18.62)	25	22.32 (13.38)	0.15 (-0.38, 0.68)	2.51
McManus et al.	2014	36	40.10 (26.40)	37	41.20 (33.70)	-0.04 (-0.49, 0.42)	2.54
Noureldeen et al.(cohort1)	2014	24	3.12 (0.30)	33	3.72 (0.32)	-1.92 (-2.56, -1.28)	2.45
Noureldeen et al.(cohort2)	2014	47	4.25 (0.43)	38	7.25 (0.82)	-4.69 (-5.53, -3.85)	2.33
Fruscalzo et al.	2015	8	0.15 (0.15)	10	0.17 (0.13)	-0.14 (-1.07, 0.79)	2.27
Saini et al.	2015	30	42.96 (19.47)	60	16.53 (7.58)	2.05 (1.52, 2.58)	2.51
Martino et al.	2016	25	27.92 (19.30)	110	21.74 (19.54)	0.32 (-0.12, 0.75)	2.55
Fatima et al.	2017	208	20.38 (12.70)	300	3.41 (2.17)	2.04 (1.83, 2.26)	2.62
Zhang et al.	2017	50	9.24 (1.94)	50	5.28 (1.29)	2.39 (1.87, 2.90)	2.52
Al-Daghri et al.	2018	63	1.58 (2.82)	54	1.37 (2.38)	0.08 (-0.28, 0.44)	2.58
Mosavat et al.	2018	53	19.80 (1.70)	43	26.60 (2.70)	-3.06 (-3.66, -2.46)	2.48
Bawah et al.	2019	70	35.04 (8.70)	70	21.94 (9.19)	1.46 (1.08, 1.83)	2.57
Ebert et al.	2020	74	26.78 (16.33)	74	23.39 (9.00)	0.26 (-0.07, 0.58)	2.59
Schuitemaker et al.	2020	50	1.44 (0.28)	100	1.32 (0.24)	0.47 (0.13, 0.81)	2.58
Wan-Qing Xiao et al.	2020	198	19.48 (9.91)	192	15.91 (9.81)	0.36 (0.16, 0.56)	2.62
Al-Musharaf et al.	2021	99	2.60 (4.60)	133	3.60 (5.10)	-0.20 (-0.46, 0.06)	2.61
Subgroup, DL		1960		3186		0.28 (-0.08, 0.63)	82.55
(I^2 = 96.6%, p = 0.000)							
yes							
Vitoratos et al.	2001	17	16.52 (5.07)	17	10.61 (1.47)	1.55 (0.77, 2.32)	2.37
Georgiou et al.	2008	14	26.10 (16.50)	14	21.20 (14.50)	0.31 (-0.44, 1.05)	2.39
López-Tinoco et al.	2012	56	27.37 (16.21)	48	18.72 (8.86)	0.64 (0.25, 1.04)	2.57
Boyadzhieva et al.	2013	127	54.27 (10.35)	109	20.40 (7.70)	3.66 (3.24, 4.08)	2.56
Zhang et al.	2016	40	10.57 (2.46)	240	4.71 (1.29)	3.87 (3.41, 4.34)	2.54
Tenenbaum-Gavish et al.	2020	20	3.87 (1.57)	185	1.94 (0.67)	2.41 (1.89, 2.93)	2.52
Florian et al.	2021	21	35.47 (18.37)	47	19.60 (17.21)	0.89 (0.36, 1.43)	2.51
Subgroup, DL		295		660		1.92 (0.77, 3.06)	17.45
(I^2 = 97.1%, p = 0.000)							
Heterogeneity between groups: p = 0.007							
Overall, DL		2255		3846		0.57 (0.19, 0.94)	100.00
(I^2 = 97.3%, p = 0.000)							

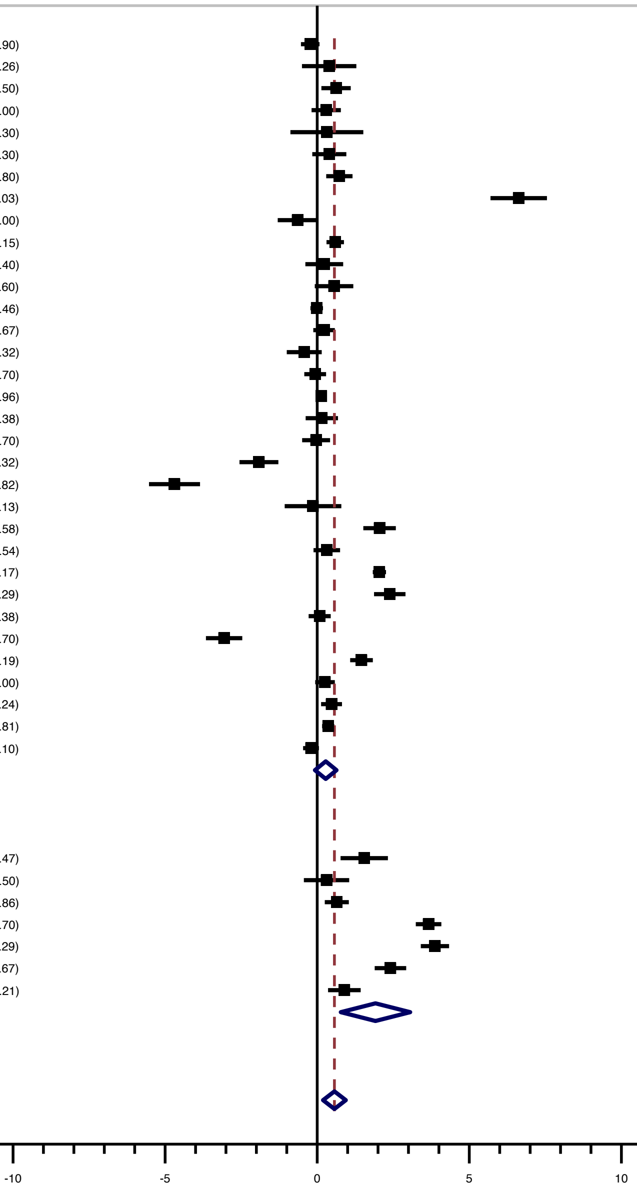

Random-effects model, inverse-variance weighting based on the DerSimonian and Laird method. Standardized mean difference (SMD) was chosen as effect size measure. A SMD>0 suggests that leptin levels are higher in GDM. Diamonds indicate the overall pooled SMDs with their corresponding 95% confidence intervals (CI).

4.10 Risk of bias

Figure S10. Forest plot graphically representing the subgroup meta-analysis evaluating the changes in circulating leptin levels between GDM patients and healthy control women, stratified by risk of bias (low vs. moderate-high).

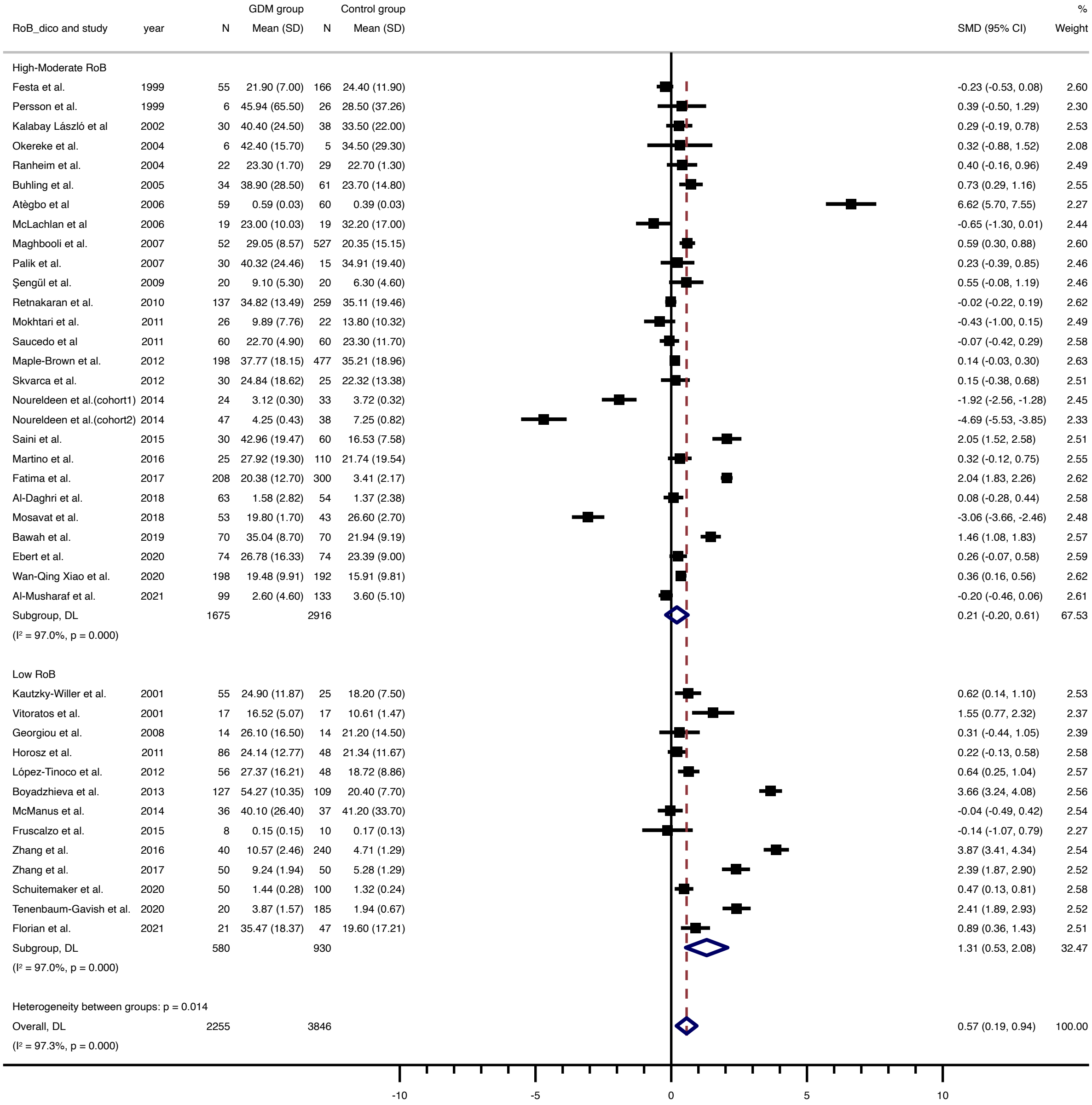

Random-effects model, inverse-variance weighting based on the DerSimonian and Laird method. Standardized mean difference (SMD) was chosen as effect size measure. A SMD>0 suggests that leptin levels are higher in GDM. Diamonds indicate the overall pooled SMDs with their corresponding 95% confidence intervals (CI).

5. Meta-regression analyses

5.1 Effect of the covariate gestational age

Figure S11. Bubble plot graphically representing the univariable meta-regression analysis of the potential effect of gestational age (weeks) on the circulating leptin levels among patients with GDM compared with healthy control women.

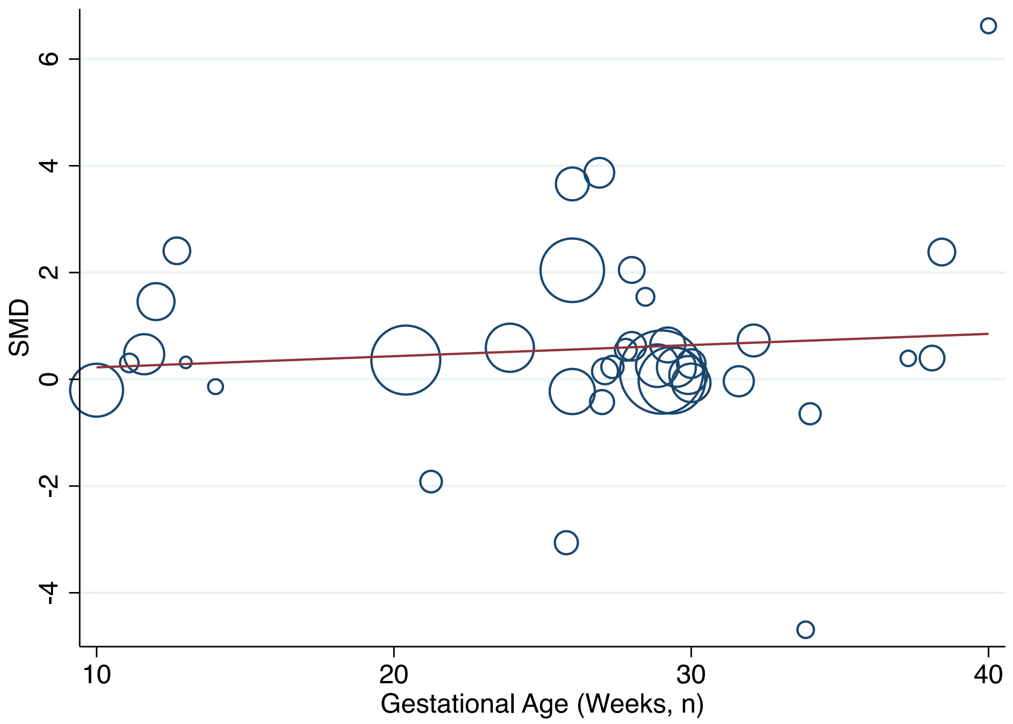

Random-effects univariable meta-regression. The red line exhibits the fitted regression line together with blue circles representing the estimates from each individual study, sized according to the precision of each estimate (the inverse of its within-study variance).

5.2 Effect of the covariate age

Figure S12. Bubble plot graphically representing the univariable meta-regression analysis of the potential effect of age (years) on the circulating leptin levels among patients with GDM compared with healthy control women.

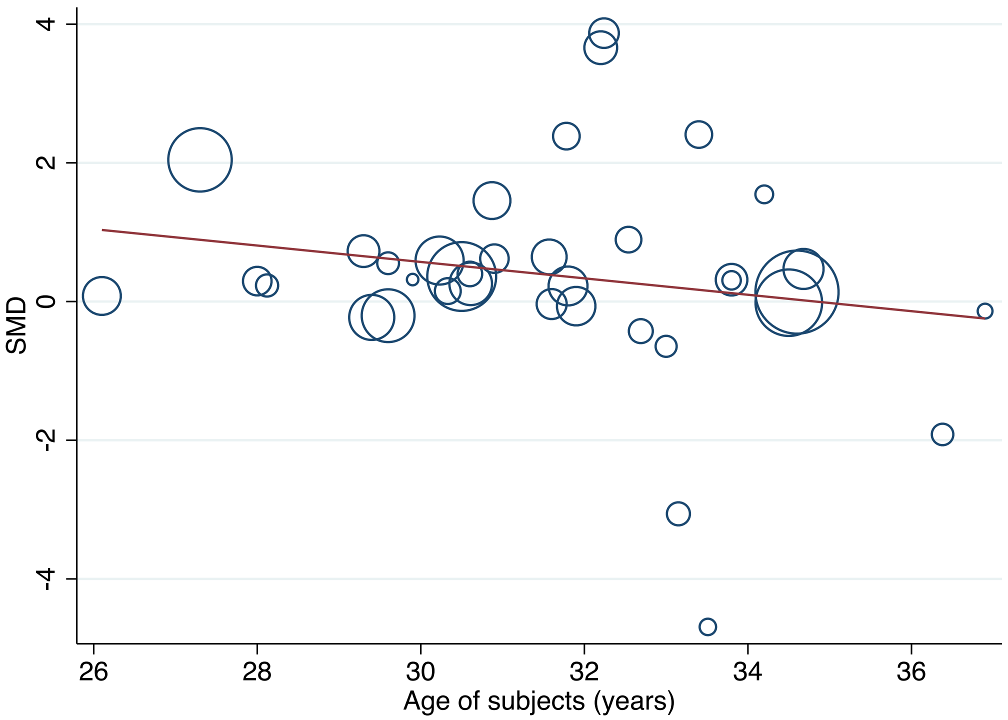

Random-effects univariable meta-regression. The red line exhibits the fitted regression line together with blue circles representing the estimates from each individual study, sized according to the precision of each estimate (the inverse of its within-study variance).

5.3 Effect of the covariate pregestational BMI

Figure S13. Bubble plot graphically representing the univariable meta-regression analysis of the potential effect of pregestational BMI (summary index score) on the circulating leptin levels among patients with GDM compared with healthy control women.

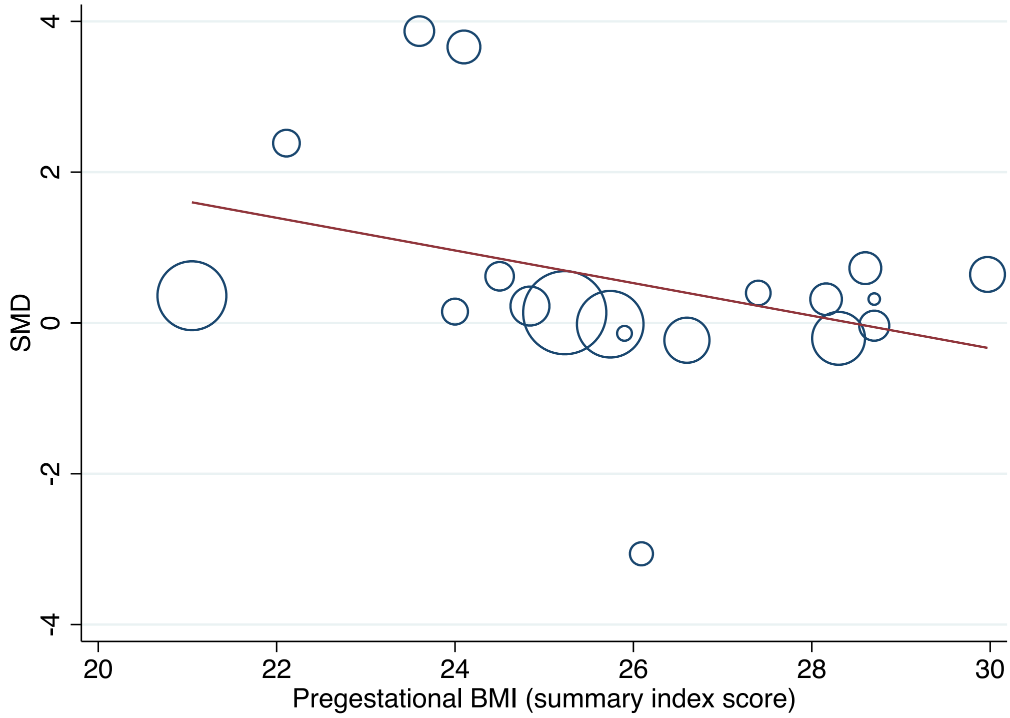

Random-effects univariable meta-regression. The red line exhibits the fitted regression line together with blue circles representing the estimates from each individual study, sized according to the precision of each estimate (the inverse of its within-study variance).

5.4 Effect of the covariate gestational BMI

Figure S14. Bubble plot graphically representing the univariable meta-regression analysis of the potential effect of gestational BMI (summary index score) on the circulating leptin levels among patients with GDM compared with healthy control women.

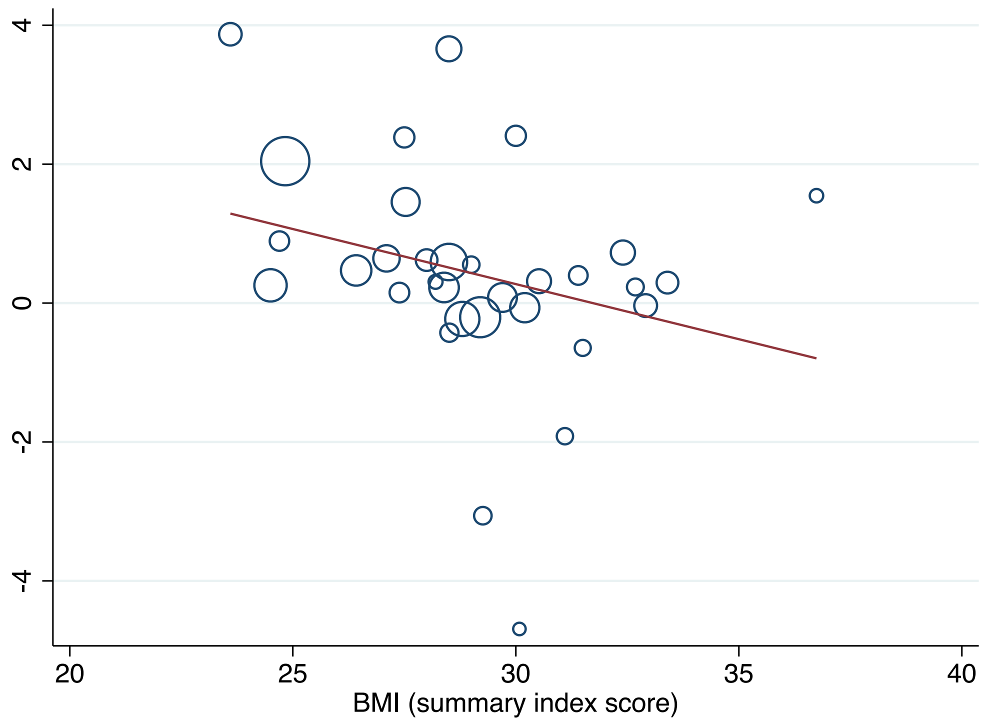

Random-effects univariable meta-regression. The red line exhibits the fitted regression line together with blue circles representing the estimates from each individual study, sized according to the precision of each estimate (the inverse of its within-study variance).

5.5 Effect of the covariate glycemia levels

Figure S15. Bubble plot graphically representing the univariable meta-regression analysis of the potential effect of glycemia levels (mmol/l) on the circulating leptin levels among patients with GDM compared with healthy control women.

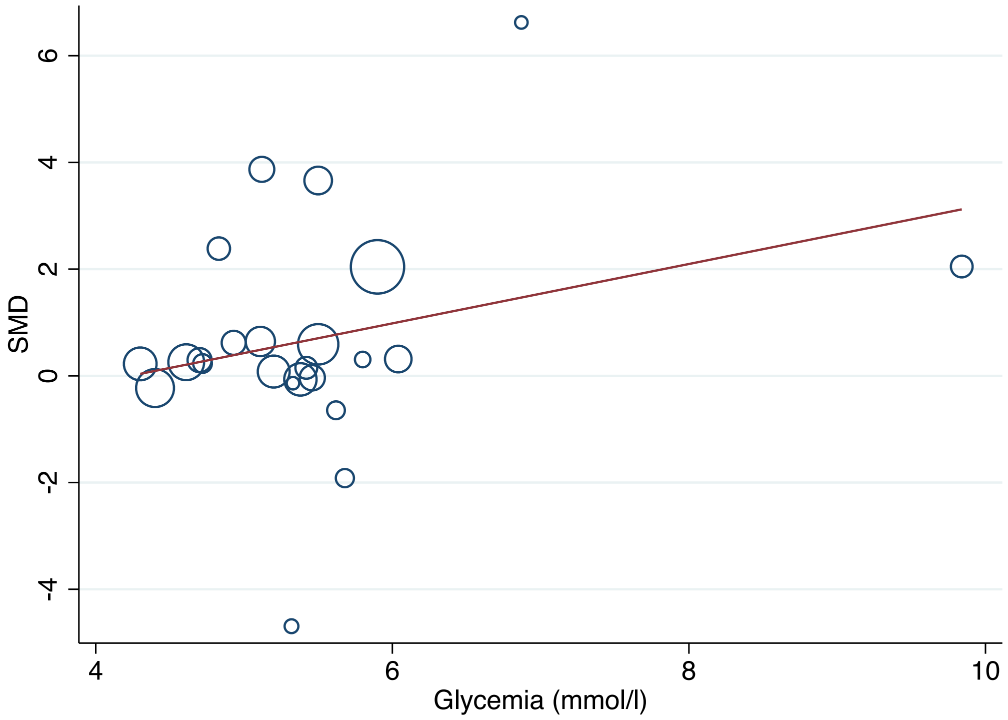

Random-effects univariable meta-regression. The red line exhibits the fitted regression line together with blue circles representing the estimates from each individual study, sized according to the precision of each estimate (the inverse of its within-study variance).

5.6 Effect of the covariate insulin

Figure S16. Bubble plot graphically representing the univariable meta-regression analysis of the potential effect of insulin (pmol/l) on the circulating leptin levels among patients with GDM compared with healthy control women.

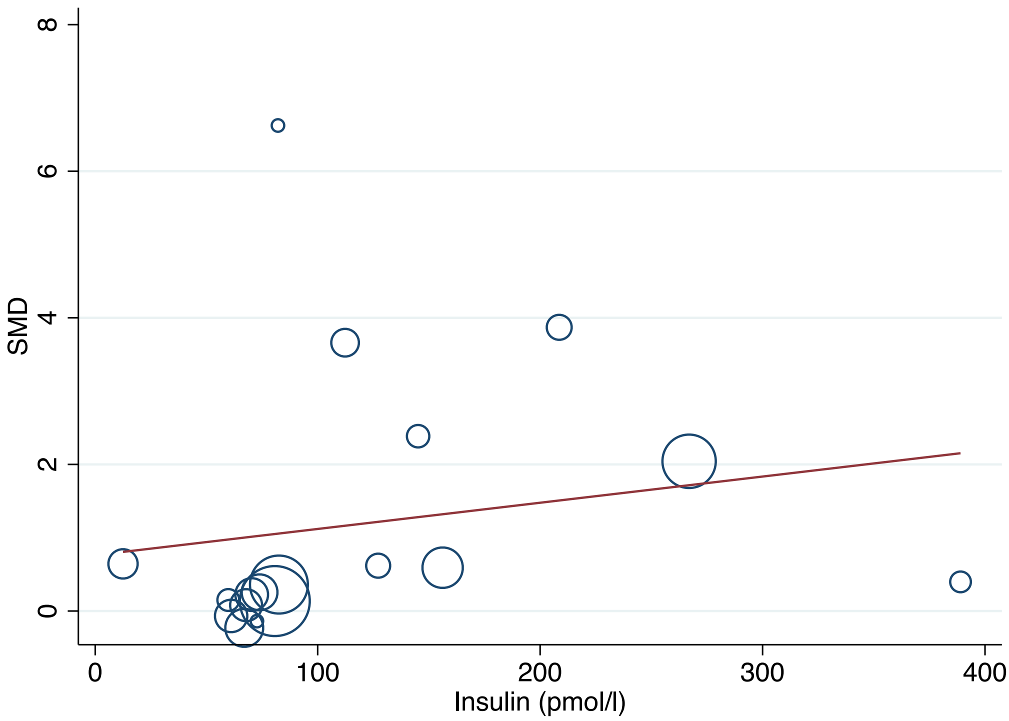

Random-effects univariable meta-regression. The red line exhibits the fitted regression line together with blue circles representing the estimates from each individual study, sized according to the precision of each estimate (the inverse of its within-study variance).

5.7 Effect of the covariate HbA1c

Figure S17. Bubble plot graphically representing the univariable meta-regression analysis of the potential effect of HbA1c (mg/dl) on the circulating leptin levels among patients with GDM compared with healthy control women.

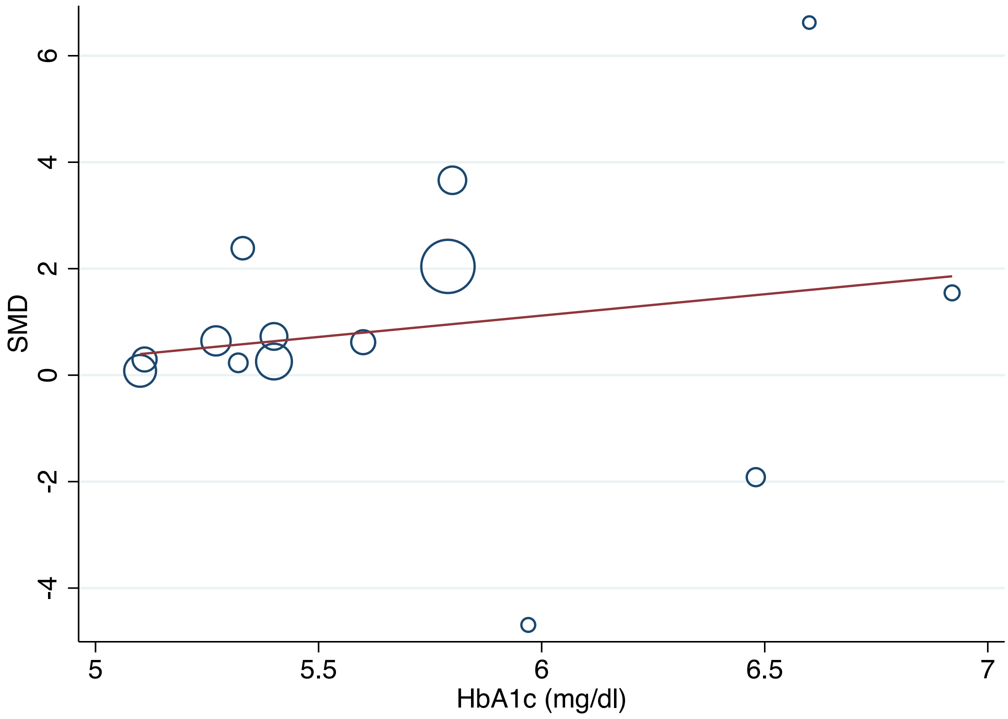

Random-effects univariable meta-regression. The red line exhibits the fitted regression line together with blue circles representing the estimates from each individual study, sized according to the precision of each estimate (the inverse of its within-study variance).

5.8 Effect of the covariate HOMA

Figure S18. Bubble plot graphically representing the univariable meta-regression analysis of the potential effect of HOMA (summary index score) on the circulating leptin levels among patients with GDM compared with healthy control women.

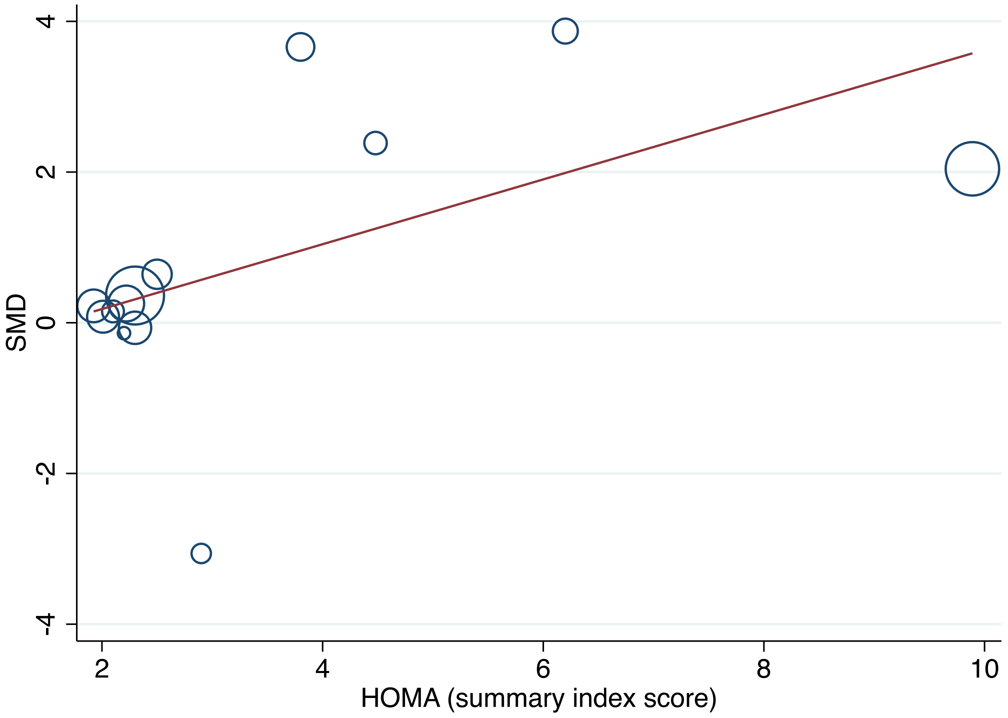

Random-effects univariable meta-regression. The red line exhibits the fitted regression line together with blue circles representing the estimates from each individual study, sized according to the precision of each estimate (the inverse of its within-study variance).

5.9 Effect of the covariate gestational age delivery

Figure S19. Bubble plot graphically representing the univariable meta-regression analysis of the potential effect of gestational age delivery (weeks) on the circulating leptin levels among patients with GDM compared with healthy control women.

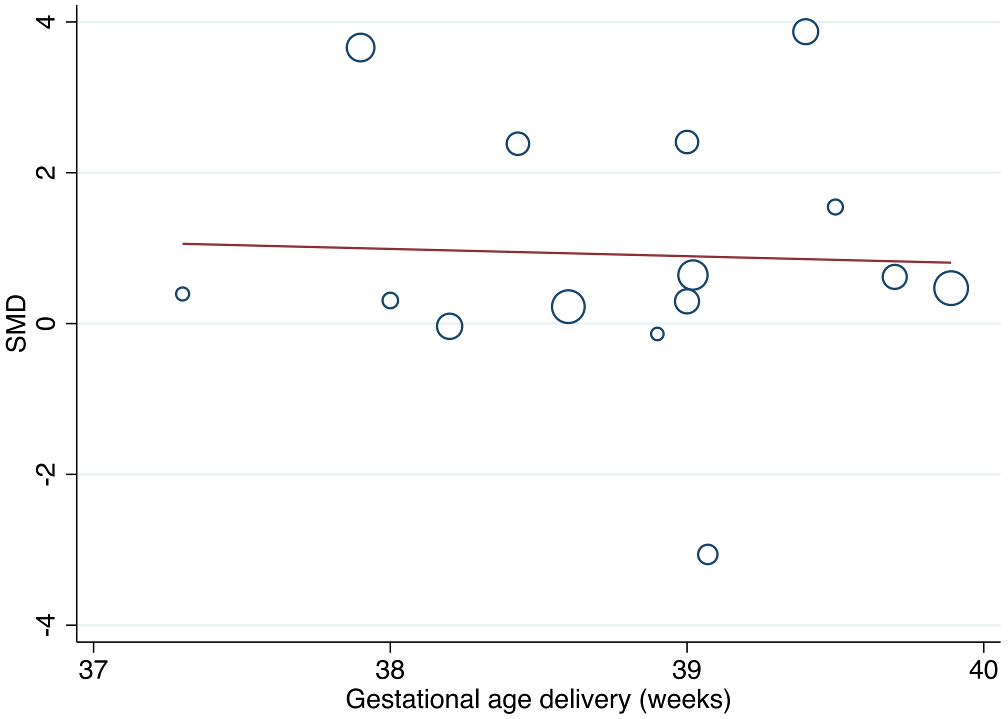

Random-effects univariable meta-regression. The red line exhibits the fitted regression line together with blue circles representing the estimates from each individual study, sized according to the precision of each estimate (the inverse of its within-study variance).

5.10 Effect of the covariate caesarian

Figure S20. Bubble plot graphically representing the univariable meta-regression analysis of the potential effect of caesarian (%) on the circulating leptin levels among patients with GDM compared with healthy control women.

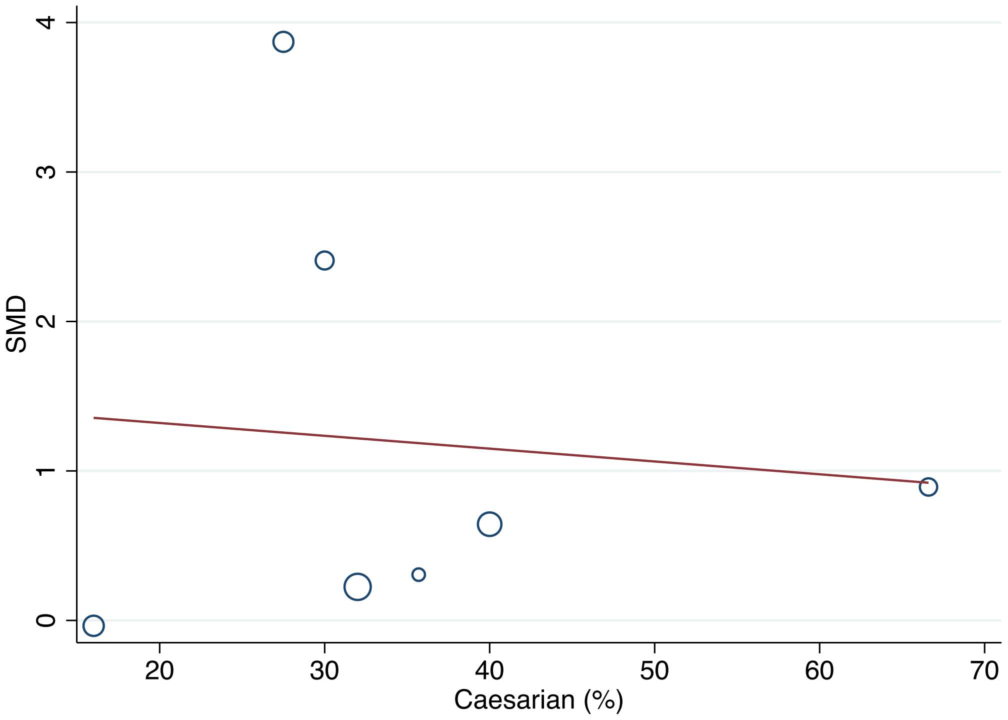

Random-effects univariable meta-regression. The red line exhibits the fitted regression line together with blue circles representing the estimates from each individual study, sized according to the precision of each estimate (the inverse of its within-study variance).

5.11 Effect of the covariate newborn weight

Figure S21. Bubble plot graphically representing the univariable meta-regression analysis of the potential effect of newborn weight (gr) on the circulating leptin levels among patients with GDM compared with healthy control women.

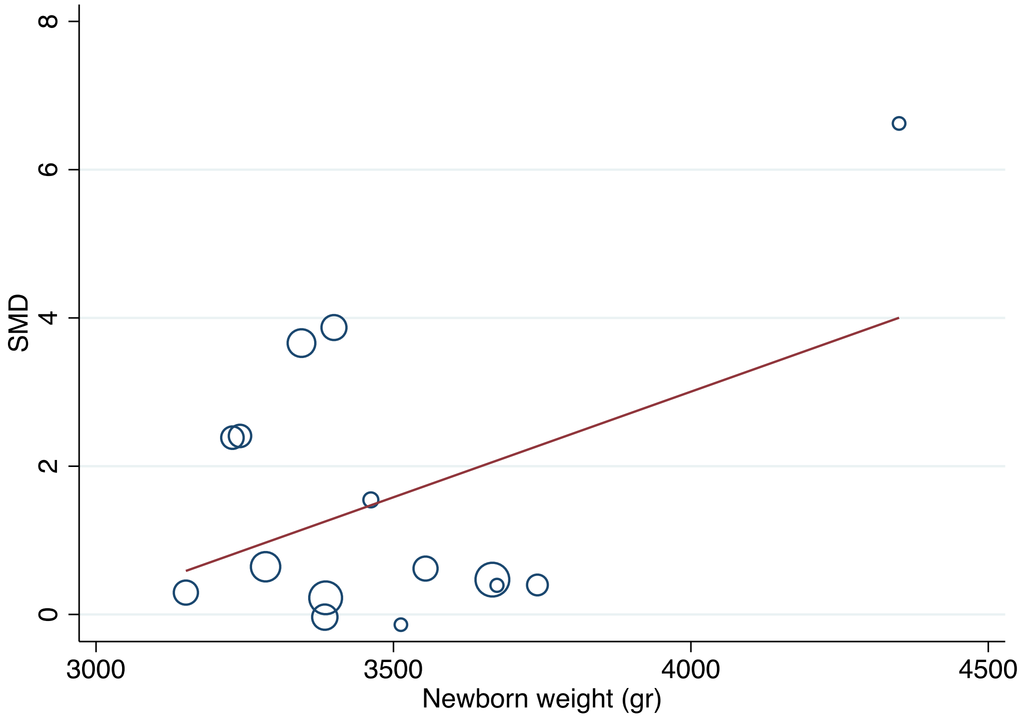

Random-effects univariable meta-regression. The red line exhibits the fitted regression line together with blue circles representing the estimates from each individual study, sized according to the precision of each estimate (the inverse of its within-study variance).

5.12 Effect of the covariate macrosomy

Figure S22. Bubble plot graphically representing the univariable meta-regression analysis of the potential effect of macrosomy (%) on the circulating leptin levels among patients with GDM compared with healthy control women.

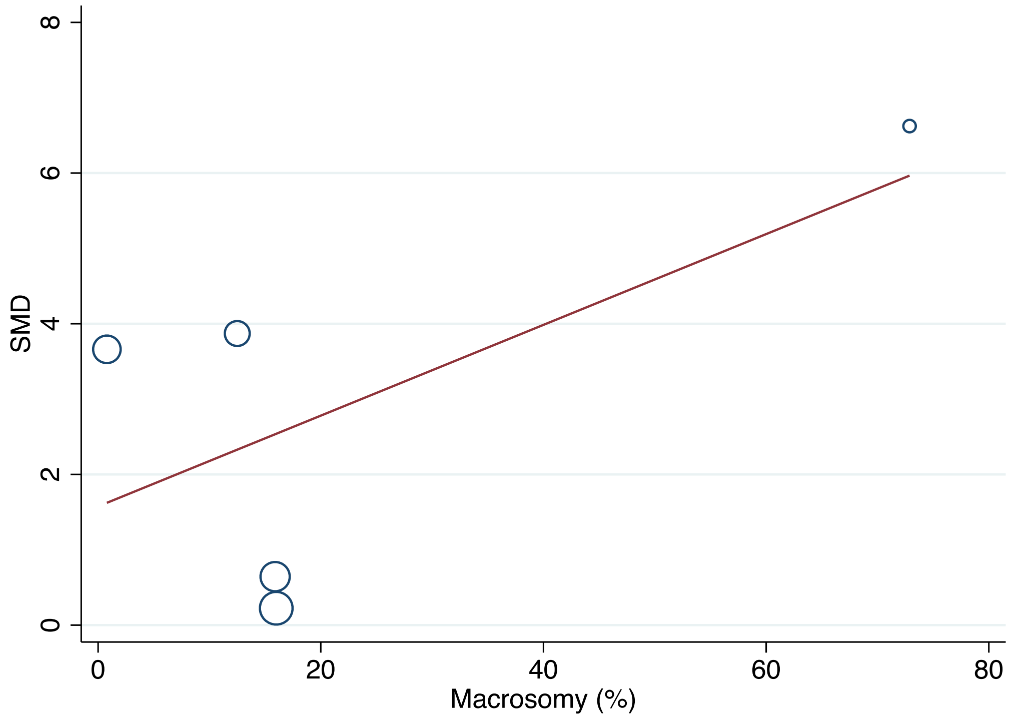

Random-effects univariable meta-regression. The red line exhibits the fitted regression line together with blue circles representing the estimates from each individual study, sized according to the precision of each estimate (the inverse of its within-study variance).

6. Small-study effects analysis

Figure S23. Funnel plot. Funnel plots of the estimated circulating leptin levels comparing GDM patients and healthy control women, expressed as standardized mean difference (SMD) against its standard error.

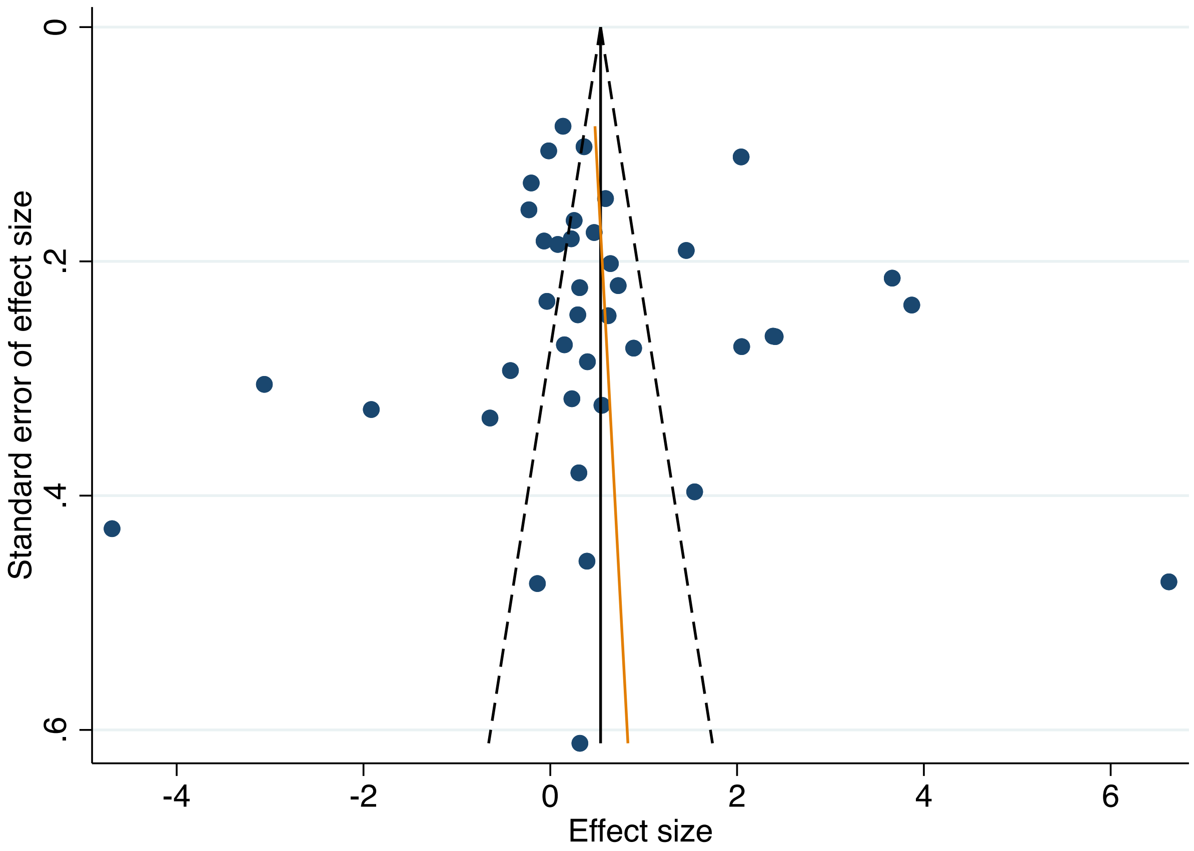

The black vertical line corresponds to the pooled SMD estimated in the meta-analysis. The two diagonal intermittent lines represent their pseudo-95%CI. The blue circles represent the estimates from primary-level studies. The orange line represents the fitted line corresponding to Egger's regression test (p=0.765) for funnel plot asymmetry.

7. Sensitivity analysis.

Figure S24. Interval plot graphically representing the sensitivity analysis ("leave-one-out" method) of the studies pooled in the meta-analysis evaluating the changes in circulating leptin levels between GDM patients and healthy control women.

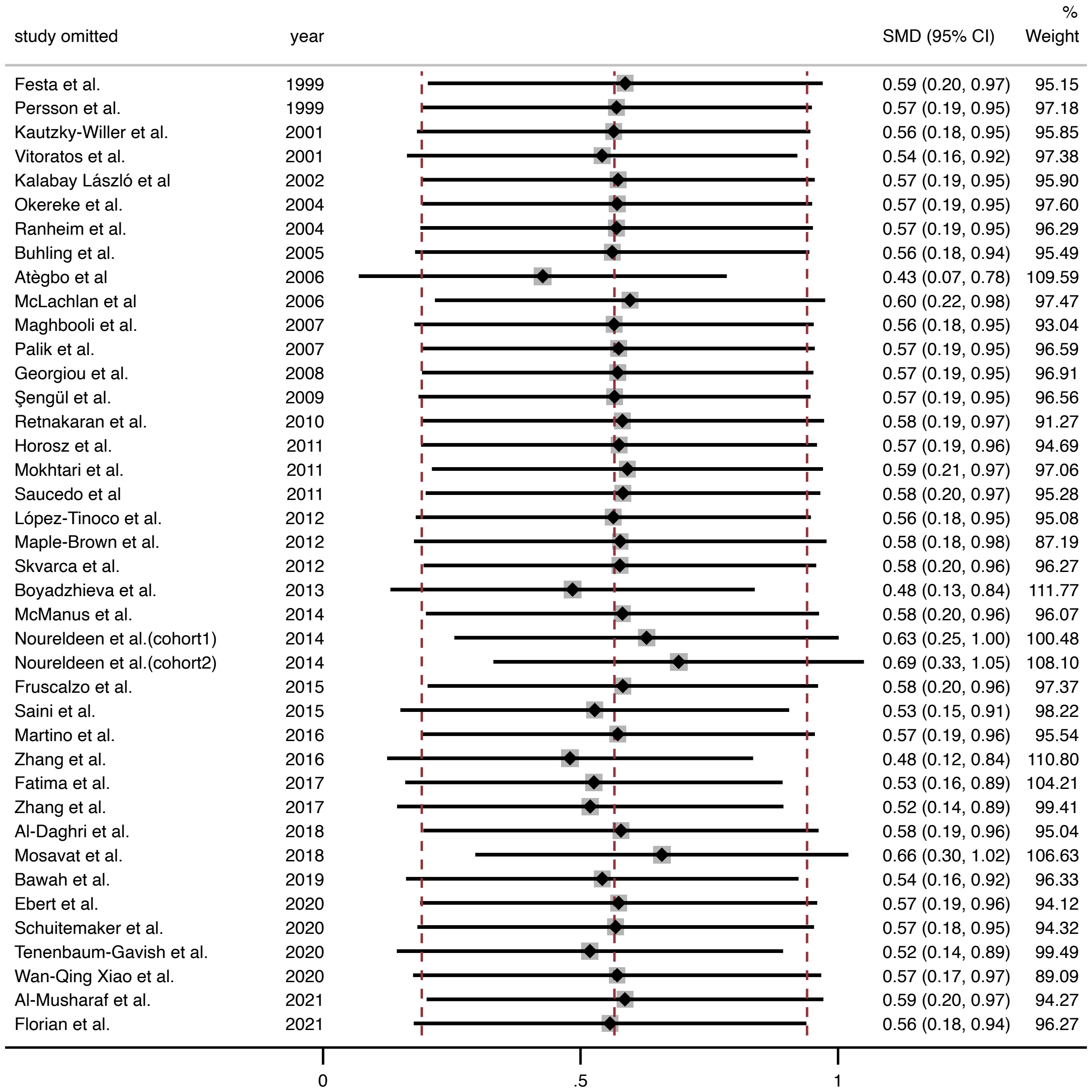

"Leave-one-out" method, sequentially omitting one study in the meta-analysis at a time, to investigate its influence on the overall result. In the interval plot, the usual diamond shape representing the pooled effect was replaced by vertical intermittent red lines, allowing a visual inspection analysis of influence.
